# Integrative taxonomy reveals a new species, *Anadara adiastola* sp. nov. (Bivalvia, Arcidae) from the northern Arabian/Persian Gulf, north-western Indian Ocean

**DOI:** 10.3897/zookeys.1294.203117

**Published:** 2026-10-13

**Authors:** Manal Al-Kandari, Matteo Garzia, Daniele Salvi, P. Graham Oliver

**Affiliations:** 1 Coastal and Marine Resources Program, Environment and Life Sciences Research Center, Kuwait Institute for Scientific Research, Hamad Al-Mubarak Street, Building 900004, Area 1, Raas Salmiya, Kuwait Coastal and Marine Resources Program, Environment and Life Sciences Research Center, Kuwait Institute for Scientific Research, Hamad Al-Mubarak Street Raas Salmiya Kuwait; 2 Department of Health, Life and Environmental Sciences, University of L’Aquila, Via Vetoio snc, 67100 L’Aquila-Coppito, Italy Department of Health, Life and Environmental Sciences, University of L’Aquila L’Aquila-Coppito Italy; 3 National Museum of Wales, Cardiff, Wales, UK National Museum of Wales Cardiff United Kingdom

**Keywords:** COI, integrative taxonomy, Kuwait, Mollusca, shell morphology

## Abstract

A new species of the arcid genus *Anadara* is described from intertidal and shallow subtidal habitats in Kuwait, northern Arabian/Persian Gulf. *Anadara adiastola***sp. nov**. is diagnosed using shell morphology and soft-part anatomy and further assessed through COI sequence data. The new species is characterised by an oblong to subtrapezoidal shell, 31–35 radial ribs, weak cross bars mainly on the anterior ribs, and a thick periostracum forming long bristles, especially on the posterior slope. This combination of characters distinguishes it from all *Anadara* species previously recorded from the Arabian/Persian Gulf, Arabian Sea, and western Indian Ocean. COI sequences from Kuwaiti specimens formed a distinct and highly divergent lineage within Anadarinae, clearly separated from all available GenBank lineages and from the regional taxa considered morphologically. The nearest molecular lineages in public databases are geographically distant and include records affected by probable misidentifications, highlighting the current limitations of GenBank for comparative taxonomy in *Anadara* while supporting the distinctiveness of the Kuwaiti lineage. The integrative taxonomic approach combining morphological, anatomical, geographic, and molecular evidence supports the recognition of *A. adiastola***sp. nov**. as a distinct species.

## Introduction

Recent surveys of the molluscan fauna of Kuwait have revealed several unresolved taxonomic problems, including an unidentified *Anadara* J.E. Gray species previously recorded as *Anadara* sp. et al. by Al-Kandari et al. (2020) and illustrated by Oliver et al. (2023; fig. 3/20). At that time, most available material consisted of disarticulated shells, while a few live collected specimens were unsuitable for molecular analysis. Later targeted collections by Al-Kandari and colleagues yielded live specimens from three localities, enabling an integrative taxonomic assessment including shell morphology, anatomy, and COI sequence data. These data support the naming of a new species from Kuwait, described below as *Anadara adiastola* sp. nov.

Although *Anadara adiastola* sp. nov. is the only living *Anadara* species found in recent surveys (Al-Kandari et al. 2020), earlier works reported several species from Kuwait. Glayzer et al. (1984) listed four species: *Anadara ehrenbergi* (Dunker, 1868), *A. antiquata* (Linnaeus, 1758), *A. uropigimelana* (Bory de St.-Vincent, 1827), and *A. secticostata* (Reeve, 1844), whereas Deeks and Jonston in Clayton (Deeks and Johnson 1983) and Jones (1986) listed and illustrated only A. ehrenbergi. Anadara (Scapharca) natalensis (Krauss, 1848) was recorded by Behbehani and Ghareeb (2002), although the specimens have not been examined by us. Based on the Smythe collection in the NHMUK, Oliver et al. (2023) found that specimens identified by Glayzer et al. (1984) as *A. antiquata* correspond to the species described here, whereas their *A. secticostata* represents A. (Mosambicarca) erythraeonensis (Jonas, 1851).

Elsewhere in the Arabian/Persian Gulf the following additional species are cited: *A. vellicata* (Reeve, 1844) (Green and Chouhfeh 1994; Oliver in Dance (Oliver 1995); *A. erythraeonensis* (Jonas in Philippi, 1851) (Al-Khayat 1997); A. (Scapharca) natalensis (F. Krauss, 1848) (Oliver in Dance et al. 1995; Al-Khayat 1997); *A. crassicostata* (H. Adams, 1873) (Huber 2010).

The Arabian/Persian Gulf shares most of its fauna with the Sea of Oman and to a considerable extent with the wider Arabian region including the Arabian Sea coasts of Pakistan and India, the northern Arabian Sea, and the Red Sea. To confirm the identity of the Kuwaiti *Anadara* species, comparisons with species from these regions are needed. Table 1 lists comparative species and additionally includes *A. auriculata* (Lamarck, 1819) (Oliver 1992), A. (Scapharca) indica (Gmelin, 1791), A. (Scapharca) inflata sensu Oliver (Oliver in Dance (Oliver 1995), as well as *A. craticulata* (Nyst, 1848), *A. gubernaculum* (Reeve, 1844), *A. inaequivalvis* (Bruguière, 1789), *A. pilula* (Reeve, 1843), *Tegillarca rhombea* (Born, 1778), and *T. granosa* (Linnaeus, 1758) (Subba Rao 2017). Taxonomic reviews of *Anadara* in the wider Western Indian Ocean are scarce. However, Lutaenko (2006) provided information for south Indian species, and Kilburn (1983) covered South African species. Huber’s (2010) general work is useful for listing described species but mainly shows images of non-type specimens. Huber’s (2010) lack of comparative data, such as rib number and shape morphometrics makes identification challenging.

**Table 1.** Specimen information for the samples sequenced in this study, including specimen and voucher codes, taxonomic identification, locality (all Kuwait), geographical coordinates, and GenBank accession numbers.

**ID_DNA**	**Voucher**	**Locality**	**Coordinates**	**GenBank accession number**
M4-1	NMW.Z.2021.009.031/4	Al-Judailiat	29.377°N, 47.759°E	PZ507397
M12-22	NMW.Z.2021.009.032/1	Shuwaikh	29.358°N, 49.947°E	PZ507398
M12-23	NMW.Z.2021.009.032/2	Shuwaikh	29.358°N, 49.947°E	PZ507399
M12-24	NMW.Z.2021.009.032/3	Shuwaikh	29.358°N, 49.947°E	PZ507400
M12-25	NMW.Z.2021.009.032/4	Shuwaikh	29.358°N, 49.947°E	PZ507401

The subfamily Anadarinae Reinhart, 1935 is accepted by MolluscaBase (MolluscaBase Eds 2025), and Feng et al. (2015) provided molecular support indicating that this taxon is monophyletic. In contrast, Arcinae is polyphyletic (Feng et al. 2015; Combosch and Giribet 2016), highlighting unresolved systematics of the Arcoida (Oliver and Holmes 2006). Within the Anadarinae, as shown by Feng et al. (2015) and Combosch and Giribet (2016), molecular support for the generic classification is weak, especially for *Scapharca* J.E. Gray, 1847, whose assigned species fall on many disparate branches. Therefore, we do not use any available subgenera within *Anadara*.

## Materials and methods

### Materials

**Figure 1.**
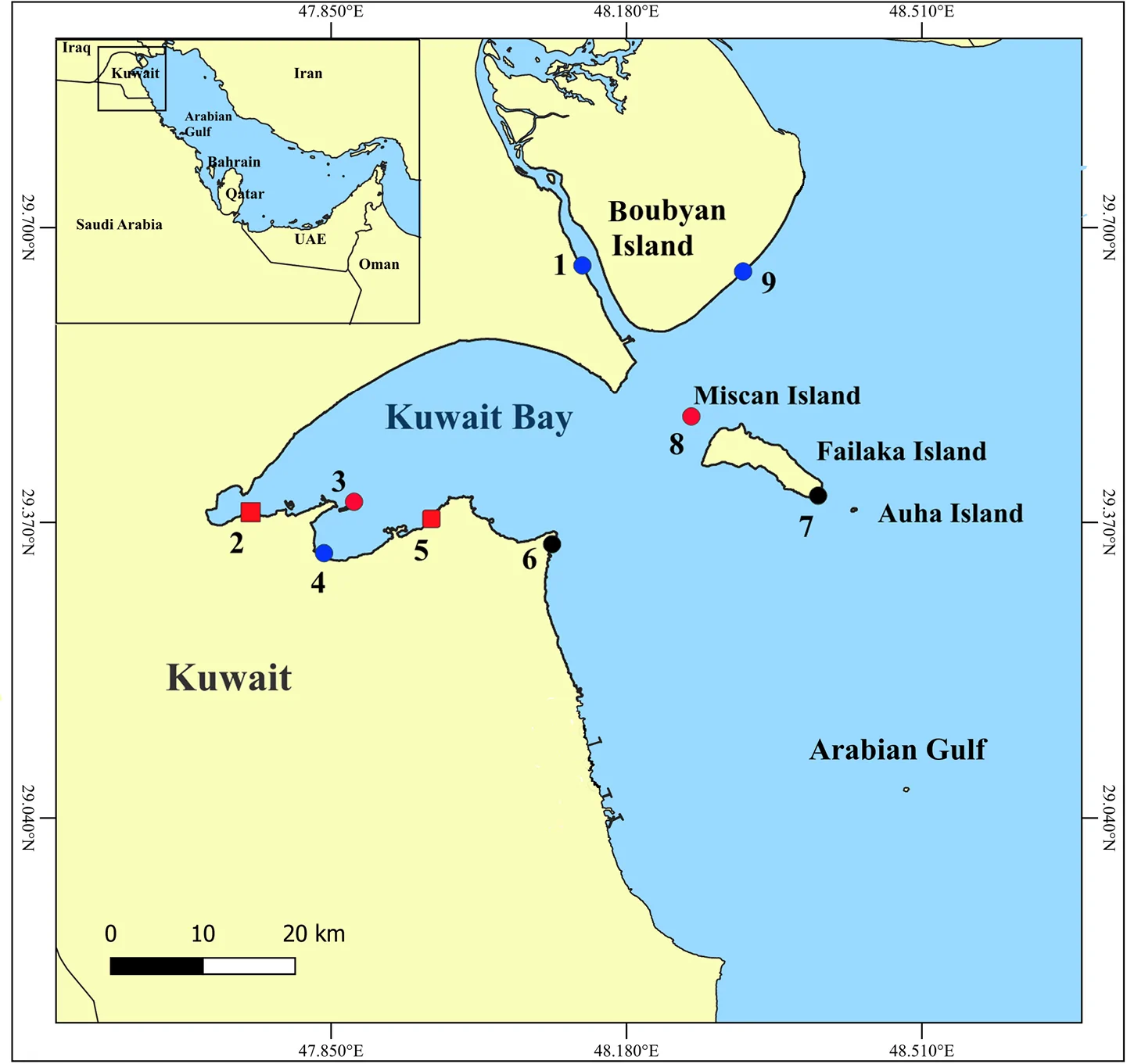
Map of northern Kuwait indicating locations where *Anadara adiastola* sp. nov. was collected. Red squares sequenced specimens, red circles living, blue circles recently dead, black circles long dead. 1. Al-Shumaimah, 2. Al-Judailiat (type locality), 3. Umm Al-Namil, 4. Subailikat Bay, 5. Shuwaikh, 6. Belajat Beach, 7. Failaka Island south, 8. Miscan Island, 9. Boubiyan south.

The material from Kuwait was collected during an intertidal survey conducted by the Kuwait Institute for Scientific Research during 2015–2018 (Al-Kandari et al. 2020) and added to by the same institute in late 2019, aided by Henk Dekker and P.G. Oliver. All the material was collected by hand digging, although this was relatively unproductive, and most shells were collected as valves from the shoreline. From 2024 to 2026, Dr Al-Kandari and her team made a concerted effort to collect living specimens for DNA sequencing, which were secured from Al-Judailiat, Shuwaikh, and Miscan Island. Locations for all occurrences are indicated in Fig. 1.

All the type series, which includes GenBank vouchers, is deposited in the National Museum of Wales, Cardiff (Zoology) under the accession numbers NMW.Z.2021.009.031 to NMW.Z.2021.009.038. Unfortunately, there is no natural history museum in Kuwait to act as a local depository.

### Morphology

The shell and anatomy were studied by P.G. Oliver, with observations made using a Leica MZ12 stereomicroscope with either a macro lens or a Leica Z6 macroscope, and the resulting photographs were focus-stacked in Helicon Focus. In some instances, the anatomy was enhanced by staining with methyl-green. The measurements were made with Vernier callipers, with linear values recorded to 0.1 decimal place in millimetres.

### DNA sequencing and molecular analyses

Total genomic DNA was extracted from alcohol-preserved tissues of the fresh specimens following standard high-salt protocols (Sambrook et al. 1989). The mitochondrial gene fragment cytochrome oxidase subunit I (COI) was amplified using degenerated primers jgLCO1490 and jgHCO2198 by Geller et al. (2013), which target the same site of the universal barcode COI region (Folmer et al. 1994). PCR conditions were as follow: 94 °C (3 min); [94 °C (60 s), 48 °C (60 s), 72 °C (60 s)] × 35 cycles; 72 °C (10 min). Sequencing of PCR products was performed by the company GENEWIZ (https://www.genewiz.com). The chromatograms of each sequence were manually edited using Geneious v. 21 (Biomatters Ltd, Auckland, New Zealand).

Newly generated COI sequences were compared against the NCBI nucleotide database using the NCBI BLASTn interface (Altschul et al. 1990) in Geneious v. 21 to determine the sequence identity. To enable a comprehensive comparison with the Kuwait samples, a COI dataset was assembled including all available sequences of Anadarinae deposited in GenBank (accessed on 01/04/2026). The selection of genera within Anadarinae followed the phylogenetic framework proposed by Feng et al. (2015). A total of 581 GenBank sequences were selected after filtering out accession numbers with short fragments (<400 bp). Three additional COI sequences of *Anadara* specimens from Oman were provided by the Florida Museum of Natural History (FLMNH). These Omani specimens originate from a geographically close region to the Kuwaiti (Arabian/Persian Gulf) samples, thereby improving spatial representation provided by the GenBank dataset. Multiple sequence alignments were performed with MAFFT v. 7.450 (Katoh and Standley 2013) using the G-INS-i iterative refinement algorithm. The final dataset comprising the Kuwait, Omani, and GenBank sequences was trimmed to the standard Folmer’s fragment length. To assess the molecular placement and divergence of Kuwaiti specimens within Anadarinae, a neighbour joining (NJ) analysis was performed using MEGA 11 (Tamura et al. 2021) using the Kimura 2-parameter (K2PA) model and 1000 bootstraps (BS) replications. *Barbatia barbata* (Linnaeus, 1758) was chosen as the outgroup for the NJ analysis according to the close relationship of the genus to the subfamily Anadarinae (Feng et al. 2015). The main clades identified in the NJ tree were collapsed using a 3% genetic distance threshold to improve visualisation of both tree topology and interclade divergences. The fully resolved (uncollapsed) NJ tree is provided in Suppl. material 1.

**Table 2.** Shells measurements of holotype and all paratypes of *Anadara adiastola* sp. nov. summarised in Fig. 3.

**Shell #**	**Site**	**Length**	**Height**	**Tumidity**	**Rib number**	**Length/height**	**Length/tumidity**	**Height/tumidity**
Holotype M4-1	A- Judailiat	53.0	39.7	32.6	32	1.34	1.63	1.22
Paratype M12-22	Shuwaikh	52.6	37.9	29.5	33	1.39	1.78	1.28
Paratype M12-23	Shuwaikh	43.1	32.7	26.8	32	1.32	1.61	1.22
Paratype M12-24	Shuwaikh	49.9	37.2	28.7	32	1.34	1.74	1.30
Paratype M12-25	Shuwaikh	43.2	33.4	25.2	33	1.29	1.71	1.33
Paratype	Shuwaikh	37.1	28.8	23.1	33	1.29	1.61	1.25
Paratype	Shuwaikh	63.5	42.2	36.2	33	1.50	1.75	1.17
Paratype M4-36	A- Judailiat	47.6	35.7	29.3	32	1.33	1.62	1.22
Paratype M4-35	A- Judailiat	56.6	41.5	37.0	33	1.36	1.53	1.12
Paratype M4-34	A- Judailiat	41.0	31.0	25.1	33	1.32	1.63	1.24
Paratype	A- Judailiat	44.0	33.4	26.2	34	1.32	1.68	1.27
Paratype	Khor Al-Sabiyah	58.7	43.1	38.5	33	1.36	1.52	1.12
Paratype	Khor Al-Sabiyah	54.9	38.3	32.5	35	1.43	1.69	1.18
Paratype	Khor Al-Sabiyah	48.9	35.4	32.2	34	1.38	1.52	1.10
Paratype	Khor Al-Sabiyah	60.0	41.5	34.4	34	1.45	1.74	1.21
Paratype	Miscan Is.	42.7	29.5	23.1	34	1.45	1.85	1.28
Paratype	Miscan Is.	36.8	27.2	23.0	33	1.35	1.60	1.18
Paratype	Miscan Is.	32.7	23.3	19.6	34	1.40	1.67	1.19
Paratype	Miscan Is.	30.9	21.8	17.7	35	1.42	1.75	1.23
Paratype	Miscan Is.	55.9	41.6	36.6	35	1.34	1.53	1.14
Paratype	Miscan Is.	50.1	38.0	32.4	34	1.32	1.55	1.17
Paratype	Miscan Is.	50.1	37.0	32.2	33	1.35	1.56	1.15
Paratype	Miscan Is.	50.1	38.0	32.4	34	1.32	1.55	1.17
Paratype	Miscan Is.	50.1	37.0	32.2	33	1.35	1.56	1.15
Paratype	Umm Al-Namil	66.1	47.3	39.7	33	1.40	1.66	1.19
Paratype	Umm Al-Namil	59.6	41.1	35	33	1.45	1.70	1.17
Paratype	Al-Doha	69.0	50.1	42.3	33	1.38	1.63	1.18
Paratype	Al-Doha	51.2	41.0	34.2	32	1.25	1.50	1.20

## Results

COI sequences were obtained from all five Kuwait specimens (GenBank accession numbers Table 1). BLASTn searches against GenBank recovered *Anadara* sequences as the closest available matches, but sequence identity was low; the closest match was *A. globosa* (MN608196), with 84.1% of pairwise identity. This high divergence from all available GenBank sequences prevents species-level identification by BLASTn.

**Figure 2.**
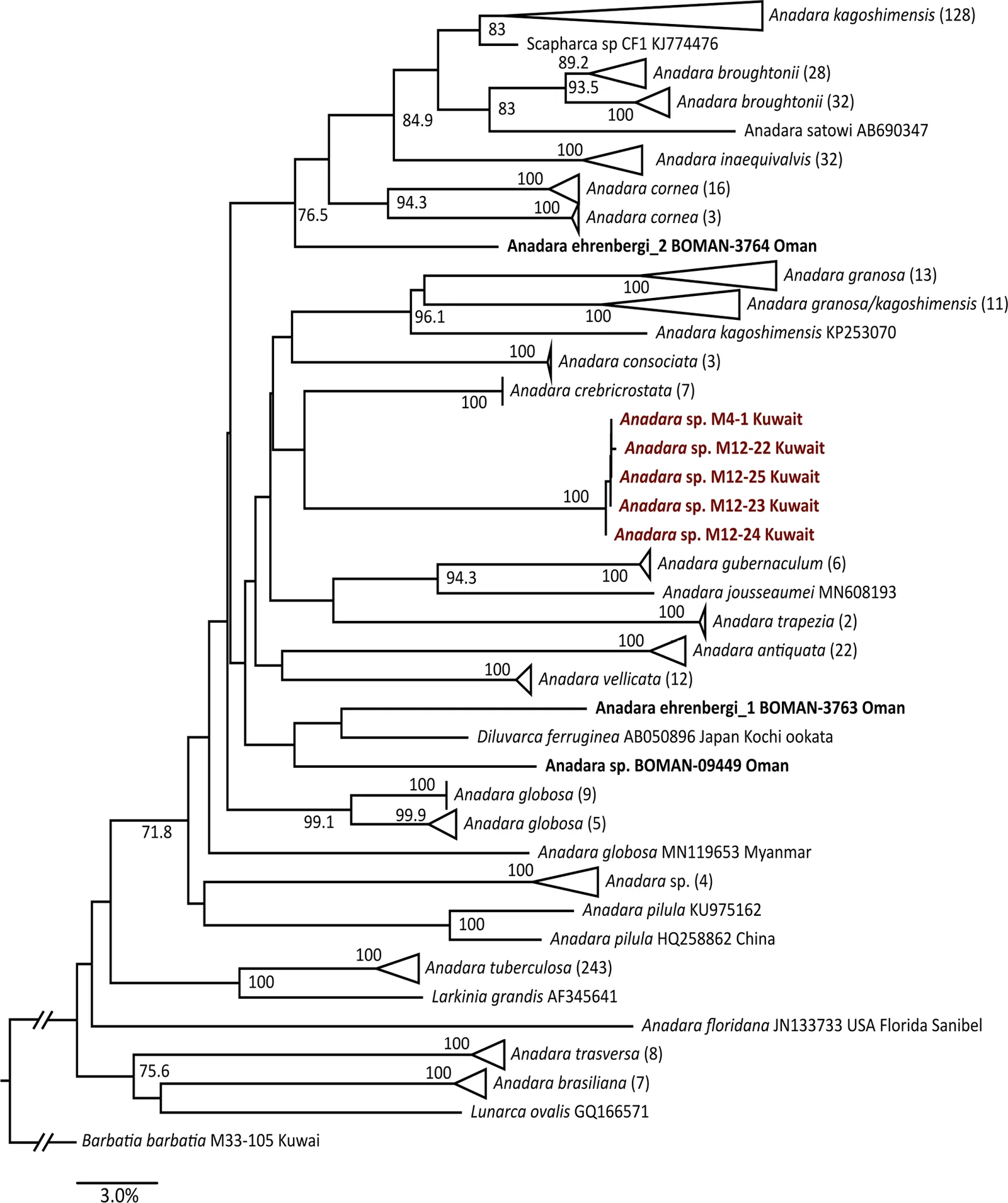
Collapsed neighbour-joining tree inferred from 588 COI sequences representing the genera *Anadara*, *Diluvarca*, *Larkinia*, *Lunarca*, *Scapharca*, and *Tegillarca*. Bootstrap support values (%) are shown only for nodes with support greater than 70%.

The NJ analysis included 588 COI sequences from *Anadara*, *Diluvarca* Woodring, 1925; *Larkinia* Reinhart, 1935; *Lunarca* J.E. Gray, 1842; *Scapharca*, and *Tegillarca* Iredale, 1939 (Fig. 2). The Kuwaiti specimens formed a distinct clade within Anadarinae and showed deeply divergence from all other sampled lineages. Their closest clade to the Kuwait specimens in the NJ tree included sequences assigned in GenBank to *A. crebricostata* Reeve, 1844, from which the Kuwaiti specimens differed by 18.7% K2P distance. However, this relationship lacked bootstrap support (BS < 70%). The Omani specimens also represented divergent lineages: the two specimens morphologically identified as *A. ehrenbergi* differed from each other by 24.6% K2P distance, whereas the third Omani *Anadara* sp. differed by 17.9% and 22.6%, respectively, from the two *A. ehrenbergi* specimens. These results indicate that the Kuwaiti specimens represent a distinct mitochondrial lineage compared to all sampled GenBank Anadarinae, highlighting the taxonomic uncertainty in GenBank records. The taxonomic implications of our results will be discussed below.

**Figure 3.**
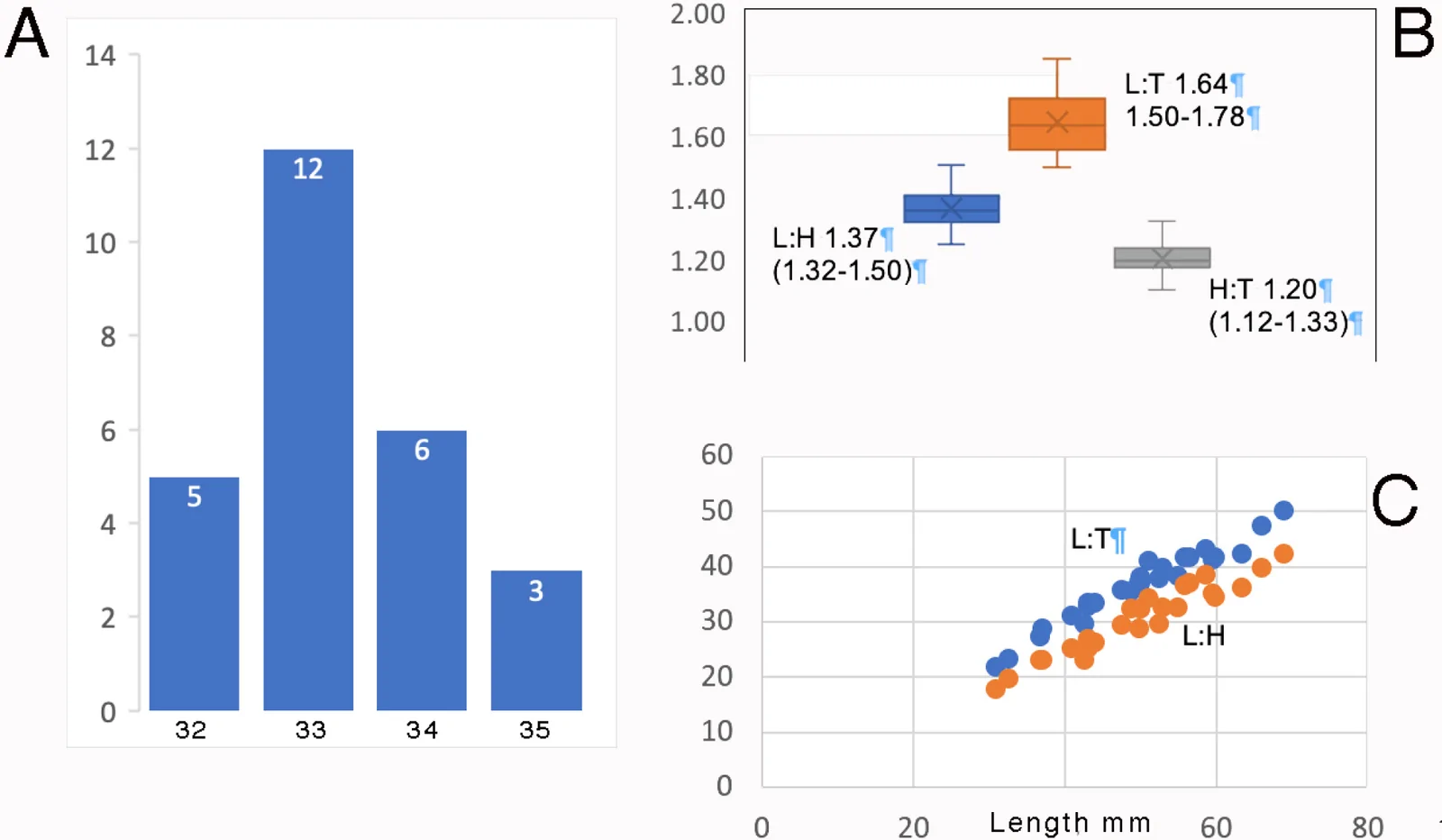
Graphical representation of morphological features of the shell of *Anadara adiastola* sp. nov. **A**. Histogram, frequency of rib number; **B**. Box-plots of the ratios length/height, length/timidity, and height/timidity, including mean values and ranges; **C**. Scatter plots of height and tumidity plotted against length.

### Systematics

**Arcoidea Lamarck, 1809**

**Arcidae Lamarck, 1809**

**Anadarinae Reinhart, 1935**

***Anadara* J.E.Gray, 1847**

#### Generic definition

The genera within the Anadarinae have never been adequately reviewed, and this is apparent in the synonymy found in MolluscaBase, where various taxa are either accepted or regarded as subgenera or as synonyms of *Anadara*. This is nowhere more apparent than in the use of *Scapharca* as a genus or subgenus. *Scapharca* has been widely used to include any *Anadara* that is inequivalve, but, as Kilburn (1983) rightly pointed out, this character is a continuum and is expressed in small specimens of the type species of *Anadara* itself.

Pending a broader revision of Anadarinae, we assign the new species to *Anadara* in the broad sense, based on its inflated, weakly sculptured radially ribbed shell lacking a byssus gape. The valves are oblong to squarish, and the species is interpreted as infaunal to semi-infaunal.

##### Anadara adiastola

Taxon classificationAnimaliaArcidaArcidae

Oliver sp. nov.

C80A54B6-CED7-5349-8C86-2E10FF64E9D8

https://zoobank.org/86634530-E1CC-43AD-B81A-1D14AF6ECF28

Figs 4, 5, 6

###### Etymology.

The specific epithet *adiastola* is derived from the Greek *adiastolos*, meaning confused or indistinct, and refers to the previous taxonomic uncertainty surrounding this species.

###### Type material.

***Holotype***: live-collected, sequenced, sequence code M4-1; Kuwait • Al-Judailiat; 29.377°N, 47.759°E; lower shore in sandy mud; 15-xii-2025; Lujain Alsayegh, Fatma AlSawagh & Lulwah Aldabbous leg.; NMW.Z.2021.009.031/4. ***Paratypes***: 3 live-collected specimens, tissues taken but not sequenced, M4B-34, MB4-35, M4B-36; same data as holotype; NMW.Z.2021.009.031/1/2/3. • 4 live-collected specimens, sequenced, sequence codes M12-22, M12-23, M12-24, M12-25; Kuwait, Shuwaikh; 29.358°N, 49.947°E; lower shore in sandy mud; 30-vi-2025; Manal Al-Kandari leg.; NMW.Z.2021.009.032/1/2/3/4. • 2 live-collected specimens, for anatomy: Kuwait, Shuwaikh; 29.358°N, 49.947°E; lower shore in sandy mud; 12-x-2025; Lulwah Aldabbous and Ali Alajmi leg.; NMW.Z.2021.009.033. • 1 shell; Kuwait, Boubyan south shore; 29.624°N, 48.299°E; strandline; 2016; PG Oliver leg.; NMW.Z.2021.009.034. • 6 shells +16valves; Kuwait, Miscan Island; 29.487493°N, 48.246921°E; dead intertidally; 15-xii-2019; H. Dekker & PG Oliver leg.; NMW.Z.2021.009.035. • 4 shells; Kuwait, Khor Al-Sabiyah; 29.741124°N, 48.095822°E; dead intertidally; 14-xii-2019; H. Dekker & PG Oliver leg.; NMW.Z.2021.009.036. • 2 specimens live collected; Kuwait, Umm Al-Namil island; 29.385946°N, 47.870563°E; in muddy gravel at low water mark; - xi-2019; PG Oliver leg.; NMW.Z.2021.009.037. • 1 live collected specimen; Kuwait, Al-Judailiat; 29.377°N, 47.759°E; lower shore in sandy mud; - xii-2019; H. Dekker & PG Oliver leg.; NMW.Z.2021.009.038. • 2 shells; Kuwait, Al-Doha; 29.390808°N, 47.820829°E; dead intertidally; 30-iii-1998; Manaf Behbehani leg.; in coll. Behbehani.

###### Other material examined.

30+ disarticulated worn valves; Kuwait, Miscan Island; strandlines; 5-ii-2026; Manal Al-Kandari and Lulwah Aldabbous leg.; NMW.Z.2021.009.039. • 20 disarticulated valves; Kuwait, Al-Judailiat; strandlines; -xi-2019; H Dekker & PG Oliver leg.; NMW.Z.2021.009.040. • 8 disarticulated valves; Kuwait, Boubyan south shore; 29.624°N, 48.299°E; strandline. 2016; PG Oliver leg.; NMW.Z.2021.009.041. • 2 disarticulated valves; Kuwait, Balajat Beach, Salmiya; -xii-2019; H. Dekker leg. and coll. •6 disarticulated valves; Kuwait, Failaka Island, south east coast; strandline; -xii-2019; H. Dekker leg. and coll. • 2 shells +12 disarticulated valves; Kuwait, Umm Al-Namil Island; long dead in intertidal muddy sand; -xi-2019; PG Oliver leg.; NMW.Z.2021.009.042. • 2 shells + 3valves; Kuwait, Miscan Island; strandlines; 5-ii-2026; PG Oliver leg.; NMW.Z.2021.009.043.

###### Diagnosis.

Shell to 70 mm in length, inequivalve; left valve slightly larger than right valve; outline oblong, subtrapezoidal, distinctly longer than high. Hinge taxodont, with up to 60 small teeth. Sculpture of 31–35 (mode 34) radial ribs; anterior ribs with distinct cross bars. Periostracum thick, forming long bristles between ribs, especially over posterior.

###### Description.

**Figure 4.**
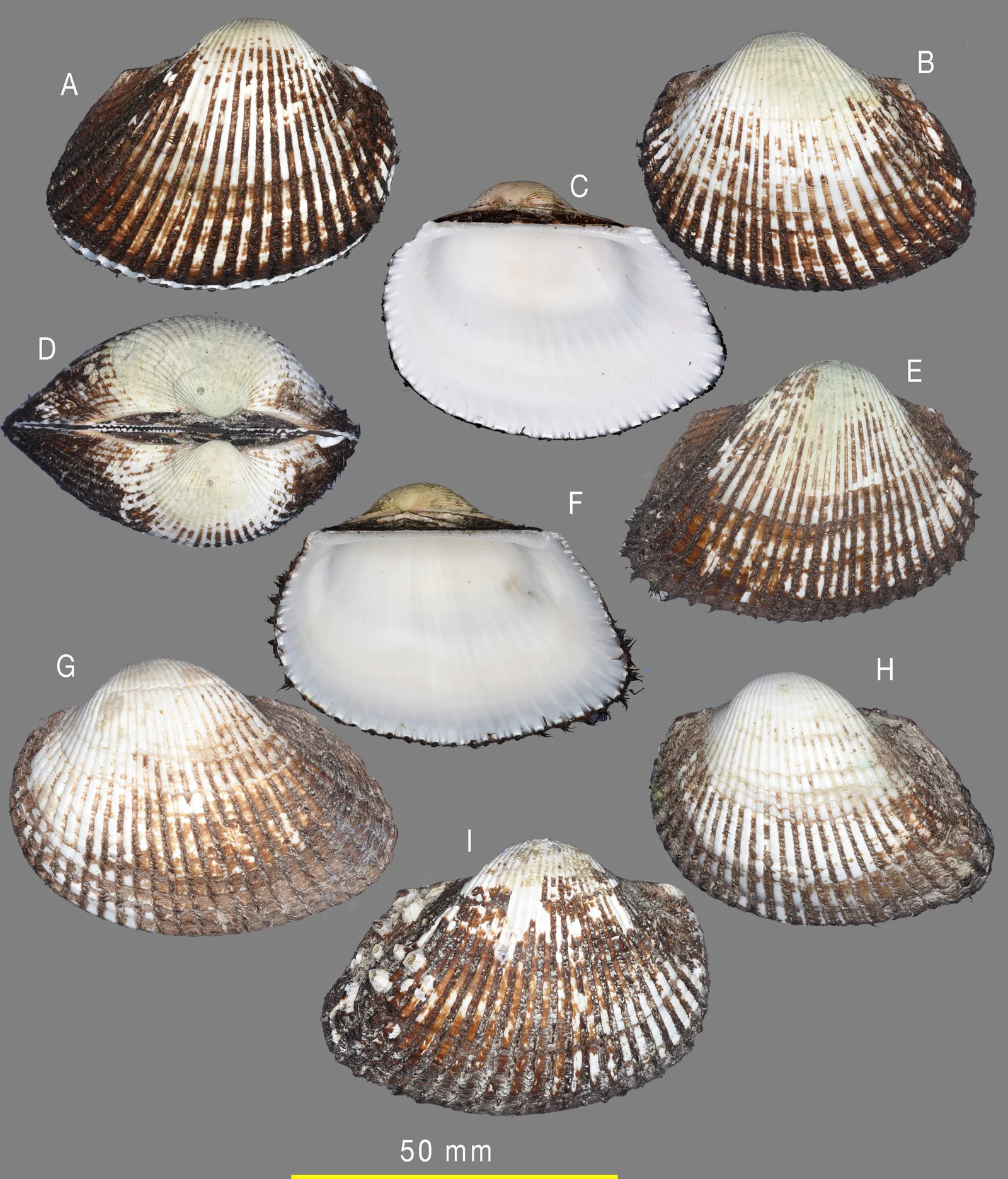
*Anadara adiastola* sp. nov. **A–D**. Holotype NMW.Z.2021.009.031/4 [M4-1], Al-Judailiat; **E, F**. Paratype NMW.Z.2021.009.031/3 [M4B-35], Al-Judailiat; **G**. Paratype, NMW.Z.2021.009.034, southern shore of Boubyan Island; **H**. Paratype, NMW.Z.2021.009.043, Miscan Island; **I**. Paratype NMW.Z.2021.009.037, Umm Al-Namil Island, live-collected specimen with epifaunal barnacles on posterior.

**Figure 5.**
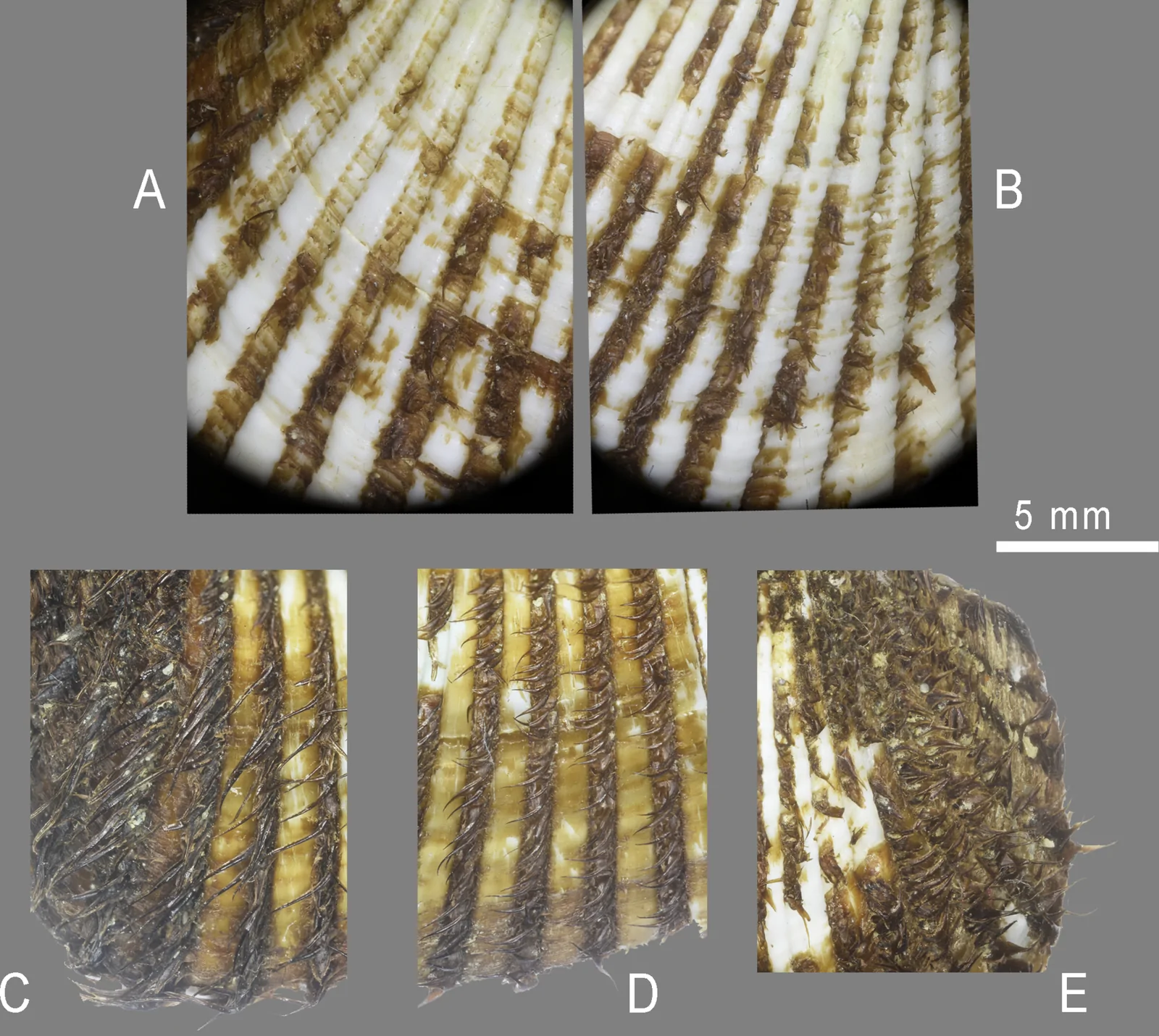
*Anadara adiastola* sp. nov. **A**. Anterior rib sculpture of left valve; **B**. Anterior rib sculpture of right valve; **C–E**. Periostracal bristles on posterior, middle, and anterior areas.

***Shell*** (Figs 4, 5). Length to 70 mm, moderately thick-shelled, tumid, almost as wide as high (height/tumidity = 1.20:1, range 1.12–1.33); inequivalve: left valve slightly larger than right valve, expressed mostly at posterior ventral margin; inequilateral: beaks in front of midline; outline oblong, subtrapezoidal, distinctly longer than high (length/ height 1.37:1, range 1.32–1.50): anterior end broadly rounded, posterior subtruncate gently convex, dorsal margin long, straight, ventral margin gently curved. Ligament of numerous chevrons, beginning in posterior but rapidly filling entire dorsal area; fibrous layers irregularly distributed. Hinge plate narrow; teeth small, numerous, increasing in number with growth, reaching a maximum number of 60; outer marginal 5 or 6 teeth largest, angled obliquely away from midline; numerous middle teeth very small, angled inward or vertical; middle edentulous space not developing. Sculpture of radial ribs numbering 31–35 (mode 34); ribs flat-topped, with rounded edges, initially equal in width to interspaces but wider than interspaces at margins in larger specimens. Ribs with weak cross bars, most developed on median anterior area of left valve, but often obscure and ribs appear smooth. Periostracum thick, olive- to chestnut-brown in colour, developed into bristles in interspaces between ribs; anterior and median bristles with short points and dorsally recurved; bristles on posterior slope much longer and ventrally projecting.

**Figure 6.**
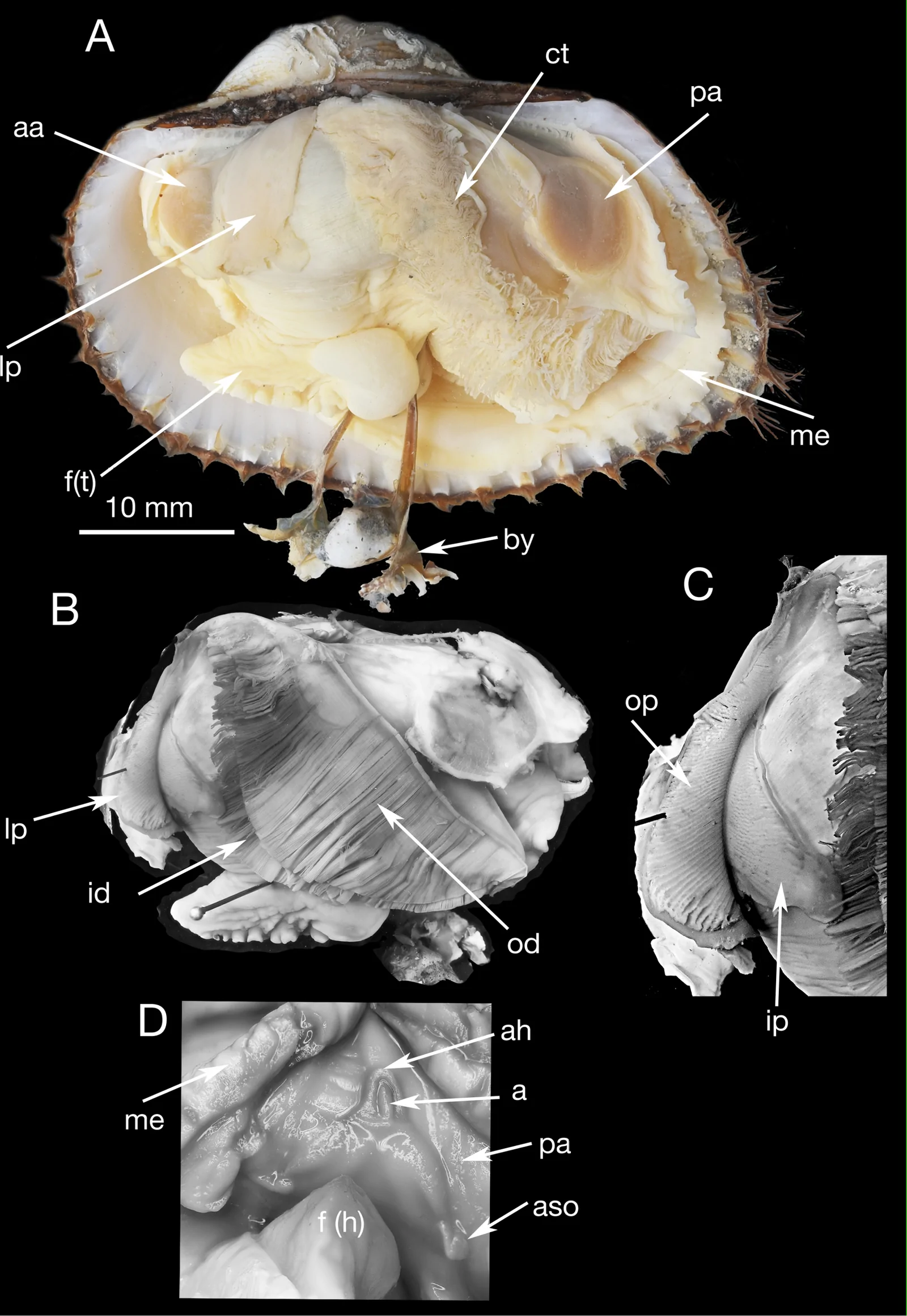
Anatomy of *Anadara adiastola* sp. nov. **A**. Gross anatomy after removal of mantle; **B**. Intact ctenidium; **C**. Detail of labial palps; **D**. Base of posterior adductor showing anus and abdominal sense organs.

***Anatomy*** (Fig. 6). Mantle edge entirely unfused; posterior inhalant aperture strongly folded, with short projecting tentacles corresponding with spaces between shell crenulations. Adductor muscles large; posterior adductor muscle slightly larger than anterior one. Foot muscular with a well-developed heel and toe; sole of toe thrown into deep folds either side of a deep groove. Byssus present in form of narrow strands adhering to sediment particles. Posterior pedal retractors and byssus retractors united, large, dorsally attached just anterior of posterior adductor muscle, attachment area roughly equal to that of posterior adductor. Ctenidia large, of two demibranchs, with outer one slightly smaller; labial palps large, with up to 82 sorting ridges on outer palp and 70 on inner palp. Rectum passes between pedal retractors and over to posterior adductor; anus surrounded by a raised ridge (hood); accessory sense organs small, as raised tubercles on ventral face of posterior adductor.

###### Comparisons.

**Figure 7.**
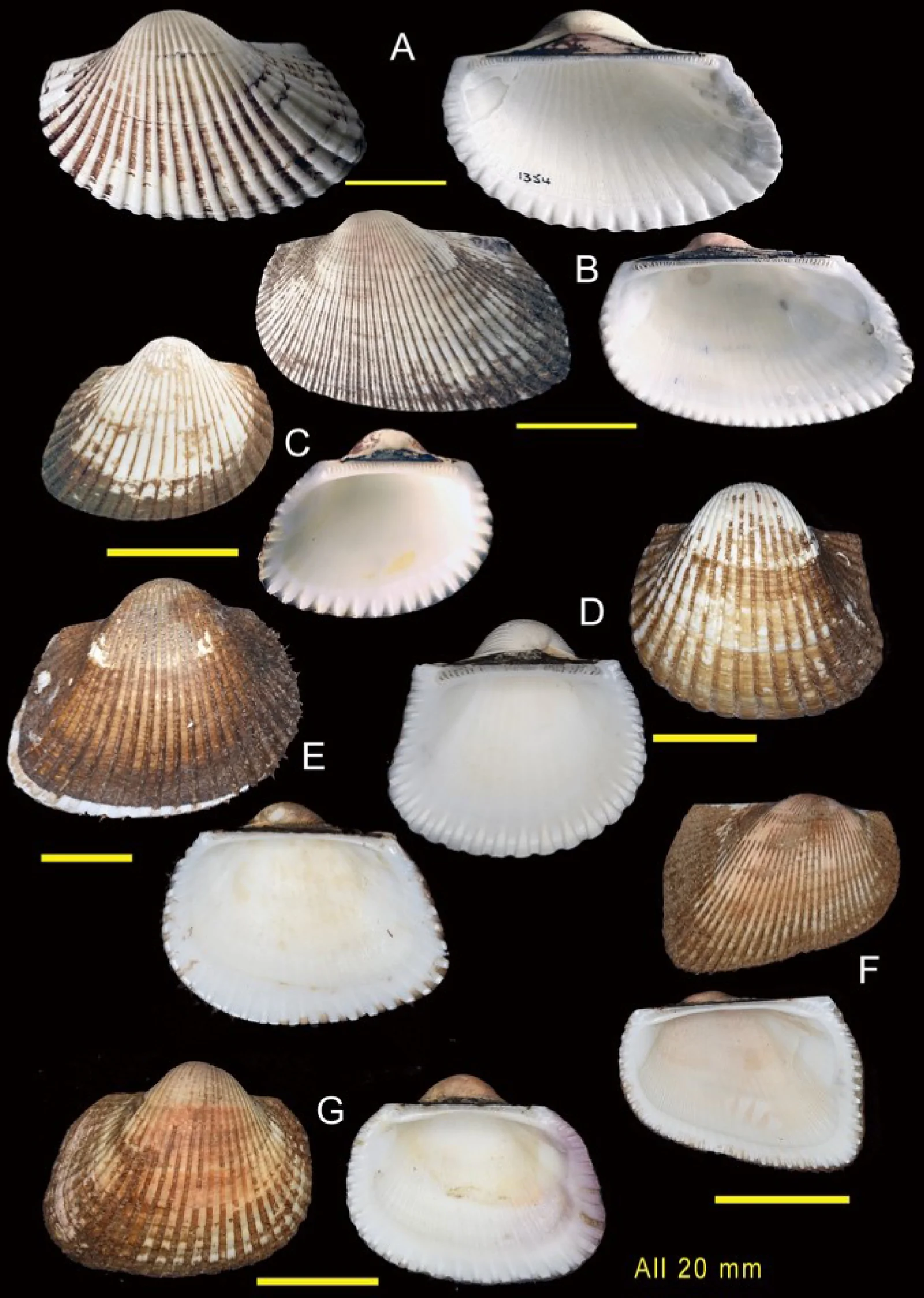
Comparative species of *Anadara*. **A**. *A. ehrenbergi*, Kuwait, Smythe Coll. NHMUK; **B**. *A. erythraeonensis*, Kuwait, Smythe Coll NHMUK; **C**, *A. uropigimelana*. Kuwait, Smythe Coll NHMUK; **D**. *A. natalensis*, Masirah, Oman, Coll. P.G. Oliver; **E**. *A. inaequivalvis*, Penang, Malaysia, NMW.Z.1955.158.10281; **F**. *A. vellicata*, Gulf of Aden, NMW.Z.1998.7.1; **G**. *A. kagoshimensis* Black Sea, leg. Anistratenko, Coll. PG Oliver.

No other species (Fig. 7) reported from Kuwait is morphologically very similar to *Anadara adiastola. Anadara ehrenbergi* is perhaps most similar but its ribs are heavier and number only 22–26 (mode 25). *Anadara erythraeonensis* has bisected ribs and *A. uropigimelana* has striate ribs. *Anadara natalensis* is quadrate in outline with 27–30 smooth ribs.

The COI data did not identify the Kuwaiti specimens with any named species currently represented in GenBank. The closest BLASTn match was *A. globosa* (MN608196), whereas the nearest clade in the NJ analysis consisted of sequences assigned to *A. crebricostata*. Both results should be interpreted cautiously because divergence values were high (>18%) and the putative relationship with the *A. crebricostata* clade was unsupported. In addition, the GenBank records assigned to *A. crebricostata* are likely misidentified, as they originate from China whereas *A. crebricostata* is endemic to Australia (Huber 2010). Lutaenko (2021) reports similar misidentifications and suggests that Chinese/Philippine records of *A. crebricostata* may correspond to *A. fultoni* (G.B. Sowerby III, 1907). Morphologically, both *A. crebricostata* and *A. fultoni* differ from *A. adiastola* sp. nov. in having bisected anterior ribs, whereas *Anadara globosa* differs in its strongly quadrate shell.

Comparisons with other species from the Arabian Gulf and Arabian Sea are provided in Table 3. Those species with a rib number that does not overlap to any degree, that is, less than 30 or more than 37 can be excluded from further comparison. This includes *A. ehrenbergi*, *A. craticulata*, *A. crassicostata*, *A. auriculata*, *A. natalensis*, *A. inflata sensu* Oliver, *A. pilula*, *A. granosa*, and *A. rhombea*.

**Table 3.** Comparative shell characters of *Anadara* species recorded from the Arabian Sea and those cited in the molecular analysis.

**Species**	**Rib number, range and (mode)**	**Rib sculpture**	**Rib divisions**	**Outline**
A. adiastola*	32–35 (34)	Weak cross bars in left valve	None	Oblong, anterior broadly rounded
A. ehrenbergi*	22–26 (25)	strong cross bars both valves	None	Oblong, anterior narrow
*A. craticulata*	22–26	Strong cross bars both valves	None	Oblong anterior narrow
*A. crassicostata*†	21–22	Weak cross lines	None	Broadly oval
*A. auriculata*	25–30	Smooth	None	Deeply oblong, posterior auriculate
*A. antiquata*†	(35)	Weak cross bars	Anterior ribs bisected	Oblique rectangular
*A. fultoni*	42–47	Weak cross bars	All ribs bisected	Oblique rectangular
*A. crebricostata*	43–44	Weak cross bars	Anterior ribs bisected	Ovate-elongate Anterior broadly rounded
A. erythraeonensis*	(36)	Weak cross bars	Most ribs bisected	Elongate oblong, anterior broadly rounded
*A. vellicata*†	(45)	Smooth	Anterior bisected, posterior striated	Trapezoidal, long posterior margin
A. natalensis*	27–30	Smooth	None	Quadrate
*A. globosa*	33–36	Smooth	None	Quadrate greatly inflated
A. uropigimelana*	(30)	Punctate	Striated ventrally	Rectangular
*A. inaequivalvis*	31–36 (34)	Smooth	None	Quadrate oblique
*A. kagoshimensis*	31–37 (35)	Cross bars	None	Oblong to roundly quadrate
*A. inflata sensu* Oliver	37	Smooth	None	Quadrate
*A. indica*	(31)	weak cross bars on left valve	none	elongate, narrowly oblong
*A. pilula*	25	granular	none	quadrate
*A. granosa*	20	nodular	none	roundly oval
*A. rhombea*	25–27	nodular	none	quadrate

*Species recorded from Kuwait. †Species recorded from the Arabian/Persian Gulf.

Both *Anadara natalensis* and *A. inflata sensu* Oliver are quadrate with the length and height approximately equal. *Anadara pilula*, *A. granosa*, and *A. rhombea* are strongly nodular, much more so than the new species. *Anadara indica* is much narrower in outline and has a much thinner shell, with the periostracum not drawn out into long bristles along the posterior slope. *Anadara vellicata* (Reeve, 1844) has a wedge-like form, with a strong posterior angle and a median sulcus.

Having removed from further consideration all those species that do not overlap in radial rib number, those with dissected ribs, and those with nodular rib sculpture, only one is left with the group of species that fall within the *A. inaequivalvis* complex. The name *A. inaequivalvis* has been widely used, as evidenced in the lengthy synonymy given by Lutaenko (2006), but this has been disputed by Huber (2010), and taxa such as *A. disparilis* (Reeve, 1844), *A. rufescens* (Reeve, 1844), and *A. rhomboidalis* (Schumacher, 1817) are now accepted by MolluscaBase (MolluscaBase Eds 2025). The type localities for these are as follows: *inaequivalvis* – Coromandel (south-east coast of India) and Tranquebar (now Tharangambadi south-east India); *rufescens* – not cited; *disparilis* – not cited; *rhomboidalis* – Tranquebar. From the type figures of all three of the latter, and the type specimens of *A. rufescens* (NHMUK 1969174) and *A. disparilis* (NHMUK 1969164), all are distinctly inequivalve, more quadrate in outline than the new species, with the length to height ratios close to 1:1. While the rib number is in keeping with the new species, the ribs are smooth, and the outline is more quadrate (Lutaenko 2006; Patterson Edward et al. 2022). The only exception is the invasive *A. kagoshimensis* Tokunaga, 1906 that has colonised the eastern Mediterranean, Black Sea, and Sea of Azov. Anistratenko et al. (2014) illustrated a considerable degree of morphological variation in this invasive species in the Black Sea, which they called *A. inaequivalvis* but now considered to be *A. kagoshimensis* (Krapal et al. 2014; Strafella et al. 2018). *Anadara kagoshimensis* has finely granulate ribs over the entire shell, whereas *A. inaequivalvis* has the ribs not granulate. Given these characters, *A. adiastola* differs from the above two species in outline and rib sculpture.

The results of the COI analysis are consistent with the morphological evidence in showing that *A. adiastola* is distinct from the available sequences assigned to *A. kagoshimensis* and *A. inaequivalvis*. However, the NJ tree should not be interpreted as a robust phylogeny of Anadarinae because several GenBank records appear taxonomically inconsistent, including multiple disparate placements of sequences assigned to *A. kagoshimensis*. The molecular analysis is used here primarily to assess sequence divergence and to test whether the Kuwaiti specimens match any available COI barcodes, rather than to infer deeper phylogenetic relationships within the group.

###### Habitat and habits.

Living specimens of *A. adiastola* were collected from across Kuwait Bay to Khor Sabiyah and Boubyan Island. In all cases the sediment is muddy to sandy mud. Living animals have only been found at the low-water mark, suggesting they may be primarily a shallow sublittoral species. Frequently the umbonal area has epifauna of worm tubes and calcareous concretions attached, suggesting that when alive they burrow only shallowly, and some individuals have been found ploughing through the sediment with their umbos exposed. This semi-infaunal habit has been described for other *Anadara* species (Lim 1966), and such species also have a few-stranded byssus attached to sediment particles. The anatomy, especially the form of the labial palps reflects the muddy, heavily suspended particle load. Lim (1966) recorded 40 and 43 ridges in *A. antiquata* and *A. anomala* (Reeve, 1844) (now *A. guangdongensis* Bernard, Cai & Morton, 1993), both of which are semi-infaunal in coarse sediments. Anadara (Tegillarca) cuneata Reeve, 1844 burrows deeply in mangrove mud and has 150 palp ridges (Lim 1966). *Anadara adiastola* has around 80 ridges, many more than in other semi-infaunal species, but fewer than in the deeply burrowing *A. cuneata*. The current high turbidity conditions in Kuwait Bay may be suitable for *A. adiastola* but not for other semi-infaunal or shallow-burrowing species more accustomed to clearer water and less muddy sediments.

**Figure 8.**
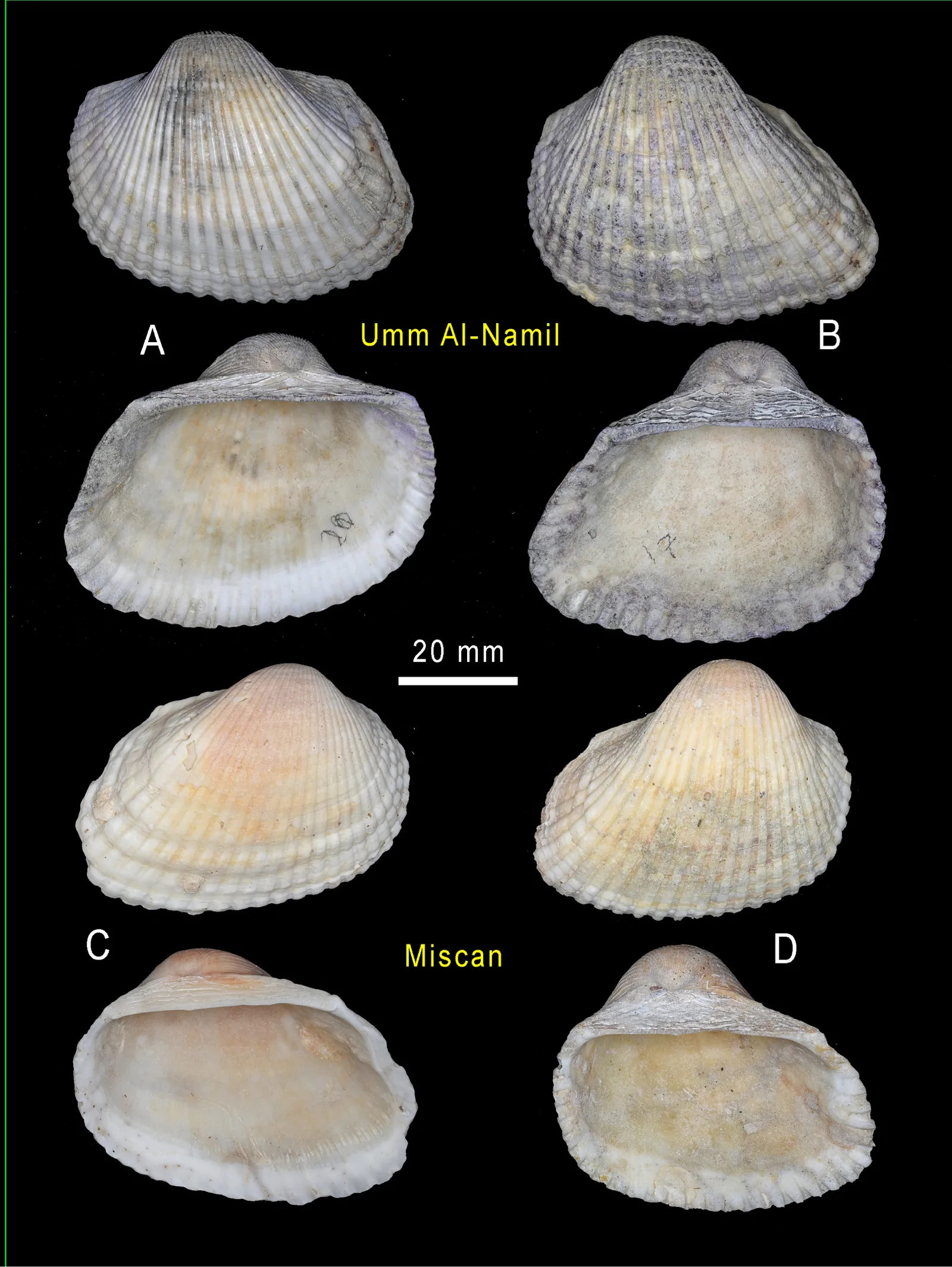
Normal compared to gerontic shells of *Anadara* from Kuwait. **A, B**. Normal (left) and umbonate (right) shells from Umm Al-Namil, NMW.Z.2021.009.042; **C, D**. Normal (left) and umbonate (right) shells from Miscan Island, NMW.Z.2021.009.039.

A few large, umbonate, gerontic shells (Fig. 8) were among the long-dead disarticulated valves collected from Miscan and Umm Al-Namil islands. These at first sight do not fit our description of *A. adiastola*. These umbonate shells (Fig. 8B, D) are large, heavy, and with about 31 ribs, although the posterior slope is rather worn. The ligament area is large and with numerous chevrons, indicating that the shells are old. Allometric growth of the ligament area has been recorded in arcoid bivalves (Thomas 1976) and in *A. diluvii* (Lamarck, 1819) in particular (Hoffman 1978). This results in a disproportionate increase with growth of the ligament area and could explain the presence of these large, umbonate shells. However, other shells typical of *A. adiastola* can reach the same size but remain unchanged in overall form (Fig. 8A, C). Allometric growth can be influenced by environmental factors such as salinity and food availability (Gosling 2015; Bullard and Roy 2025). The variations observed here may, therefore, reflect local environmental conditions affecting only some individuals. However, because other *Anadara* species have been recorded from Kuwait in the recent past (Glayzer et al. 1984) and in Holocene deposits between 6990 cal yr BP and ~3370 cal yr BP (Reinink-Smith 2015), the possibility that some large, umbonate shells represent another species cannot be excluded. At present, too few such shells are available to resolve this issue, and additional live-collected material suitable for sequencing is needed to confirm their identity.

## Discussion

### GenBank sequences

The COI data provide independent support for the distinctiveness of *Anadara adiastola* but also illustrate the limitations of public sequence databases for taxonomic revision. Reliable sequence-based identification depends on correctly identified voucher specimens. Ideally these should come from type localities or from material directly comparable with type material. In this analysis, the Kuwaiti specimens did not closely match any available GenBank sequence. The closest BLASTn match was with *A. globosa*, whereas the nearest clade in the NJ tree was composed of sequences assigned to *A. crebricostata*. Both comparisons involved highly divergent lineages, and the putative relationship with the *A. crebricostata* clade was not supported. Moreover, the sequences assigned to *A. crebricostata* are probably misidentified because they originate from China, outside the accepted Australian range of *A. crebricostata*. Similar caution applies to sequences assigned to *A. inaequivalvis* from China and Japan, whereas the type locality of that species lies in southern India. These issues do not weaken the recognition of *A. adiastola*; rather, they show that morphology, anatomy, geography, and sequence divergence must be interpreted together in this group.

### Alien/invasive status

**Figure 9.**
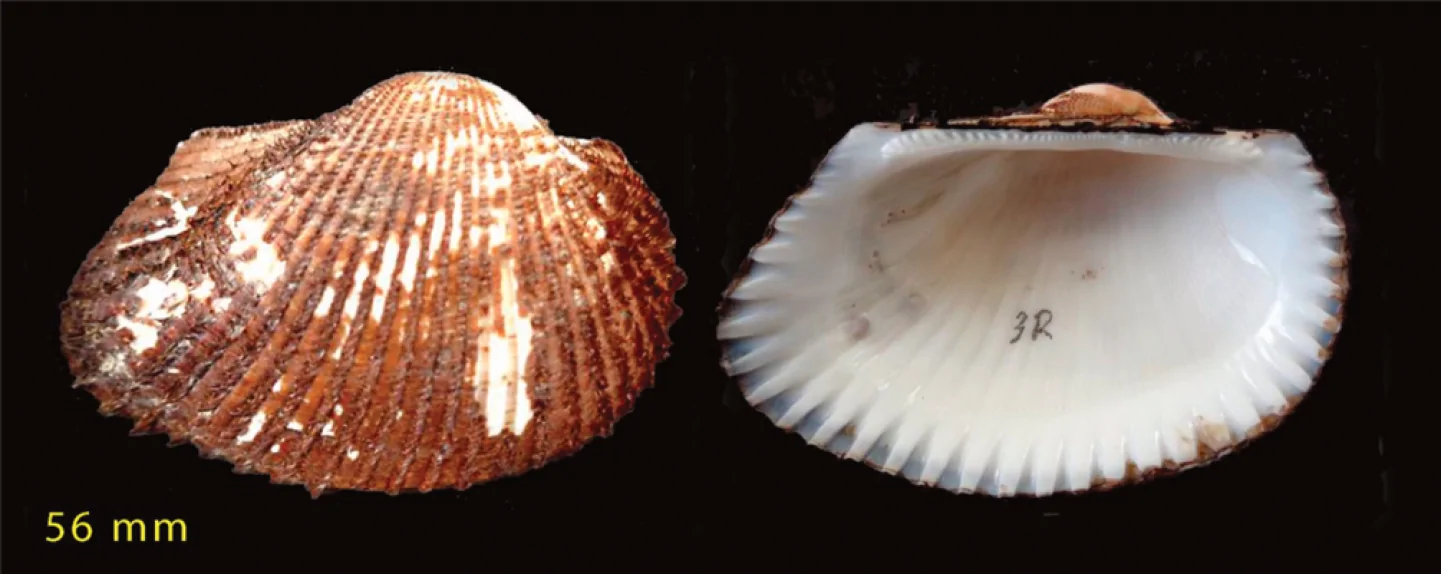
An unidentified *Anadara* from the Balochistan coast of Pakistan, leg. Saedul Bukhari.

Newly detected marine molluscs may raise questions about possible alien or invasive status, especially when locally abundant. Given the high volume of global shipping into the Arabian/Persian Gulf, an alien origin for *A. adiastola* cannot be excluded. However, if the species is non-native, its arrival is unlikely to be recent. Material attributable to this species was already present in Kuwait in the collections reported by Glayzer et al. (1984). *Anadara kagoshimensis*, *A. inaequivalvis*, and *A. natalensis* have all invaded the Mediterranean from the Indo-Pacific, but these taxa can be excluded as candidates for the Kuwaiti species on morphological grounds. Oliver et al. (2023) noted faunal affinities between Kuwait and Pakistan/north-west India, and this pattern may be relevant here. Jahangir et al. (2012) reported and illustrated *A. antiquata* from muddy creeks on the Balochistan coast of Pakistan. However, the illustrated shell does not appear to have the bisected ribs typical of that species. Images of a similar shell from Balochistan (Fig. 9), sent to P.G. Oliver, also resemble *A. adiastola* and warrant further investigation, although no specimens were available for detailed morphological examination and no tissue material could be obtained for sequencing. At present, the available evidence supports the recognition of *A. adiastola* as a distinct species in Kuwait. It remains possible that related or conspecific populations may occur elsewhere along the northern Arabian Sea coast.

## Supplementary Material

XML Treatment for Anadara adiastola

## Additional information

### Artificial Intelligence (AI) use

The authors accept full responsibility for the content of the manuscript, including the disclosure of any use of AI.

No AI tools were used in the preparation of this manuscript.

### Author ORCIDs

M. Al-Kandari https://orcid.org/0000-0003-0073-7929

M. Garzia https://orcid.org/0000-0002-0918-9925

D. Salvi https://orcid.org/0000-0002-3804-2690

P.G. Oliver https://orcid.org/0000-0002-9481-0711

### Data availability

All of the data that support the findings of this study are available in the main text or Supplementary Information.

## Supplementary materials

Supplementary material 1 Full tree from which the collapsed tree in Fig. 2 was generated Data type pdf Explanation note Neighbor-joining tree from analysis of 588 COI sequences from the genera *Anadara*, *Diluvarca*, *Larkinia*, *Lunarca*, *Scapharca*, and *Tegillarca*. Bootstrap values (%) are indicated only for nodes with support >70%. https://binary.pensoft.net/file/1807872This dataset is made available under the Open Database License (http://opendatacommons.org/licenses/odbl/1.0/). The Open Database License (ODbL) is a license agreement intended to allow users to freely share, modify, and use this Dataset while maintaining this same freedom for others, provided that the original source and author(s) are credited. Daniele Salvi, Matteo Grazia
