# Supplementary material for: Integrative taxonomy reveals a new species, Anadara adiastola sp. nov. (Bivalvia, Arcidae) from the northern Arabian/Persian Gulf, north-western Indian Ocean
Source: Zookeys. 2026 Oct 13;1294:333–53. doi: 10.3897/zookeys.1294.203117 (PMC13684320; doi:10.3897/zookeys.1294.203117)
Supplement: Supplementary file 1 [file zookeys-1294-333_article-203117__-s001.pdf]

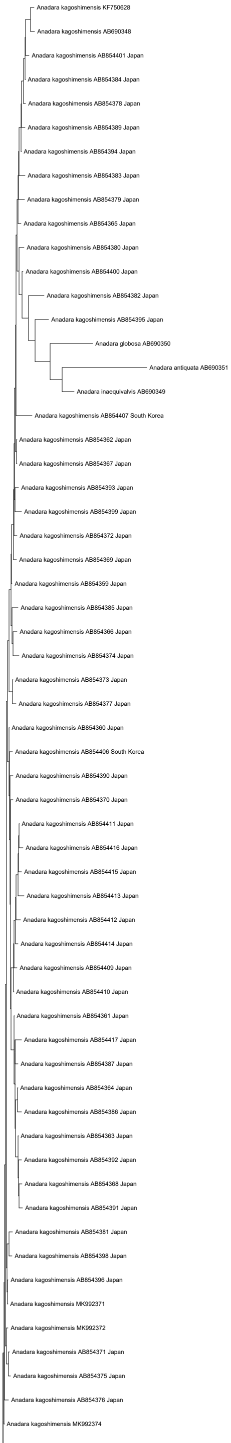
Anadara kagoshimensis KF750628
Anadara kagoshimensis AB690348
Anadara kagoshimensis AB854401 Japan
Anadara kagoshimensis AB854384 Japan
Anadara kagoshimensis AB854378 Japan
Anadara kagoshimensis AB854389 Japan
Anadara kagoshimensis AB854394 Japan
Anadara kagoshimensis AB854383 Japan
Anadara kagoshimensis AB854379 Japan
Anadara kagoshimensis AB854365 Japan
Anadara kagoshimensis AB854380 Japan
Anadara kagoshimensis AB854400 Japan
Anadara kagoshimensis AB854382 Japan
Anadara kagoshimensis AB854395 Japan
Anadara globosa AB690350
Anadara antiquata AB690351
Anadara inaequivalvis AB690349
Anadara kagoshimensis AB854407 South Korea
Anadara kagoshimensis AB854362 Japan
Anadara kagoshimensis AB854367 Japan
Anadara kagoshimensis AB854393 Japan
Anadara kagoshimensis AB854399 Japan
Anadara kagoshimensis AB854372 Japan
Anadara kagoshimensis AB854369 Japan
Anadara kagoshimensis AB854359 Japan
Anadara kagoshimensis AB854385 Japan
Anadara kagoshimensis AB854366 Japan
Anadara kagoshimensis AB854374 Japan
Anadara kagoshimensis AB854373 Japan
Anadara kagoshimensis AB854377 Japan
Anadara kagoshimensis AB854360 Japan
Anadara kagoshimensis AB854406 South Korea
Anadara kagoshimensis AB854390 Japan
Anadara kagoshimensis AB854370 Japan
Anadara kagoshimensis AB854411 Japan
Anadara kagoshimensis AB854416 Japan
Anadara kagoshimensis AB854415 Japan
Anadara kagoshimensis AB854413 Japan
Anadara kagoshimensis AB854412 Japan
Anadara kagoshimensis AB854414 Japan
Anadara kagoshimensis AB854409 Japan
Anadara kagoshimensis AB854410 Japan
Anadara kagoshimensis AB854361 Japan
Anadara kagoshimensis AB854417 Japan
Anadara kagoshimensis AB854387 Japan
Anadara kagoshimensis AB854364 Japan
Anadara kagoshimensis AB854386 Japan
Anadara kagoshimensis AB854363 Japan
Anadara kagoshimensis AB854392 Japan
Anadara kagoshimensis AB854368 Japan
Anadara kagoshimensis AB854391 Japan
Anadara kagoshimensis AB854381 Japan
Anadara kagoshimensis AB854398 Japan
Anadara kagoshimensis AB854396 Japan
Anadara kagoshimensis MK992371
Anadara kagoshimensis MK992372
Anadara kagoshimensis AB854371 Japan
Anadara kagoshimensis AB854375 Japan
Anadara kagoshimensis AB854376 Japan
Anadara kagoshimensis MK992374

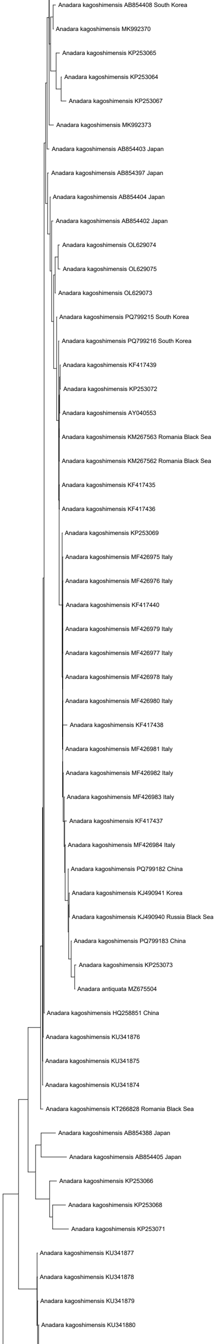
Anadara kagoshimensis AB854408 South Korea
Anadara kagoshimensis MK992370
Anadara kagoshimensis KP253065
Anadara kagoshimensis KP253064
Anadara kagoshimensis KP253067
Anadara kagoshimensis MK992373
Anadara kagoshimensis AB854403 Japan
Anadara kagoshimensis AB854397 Japan
Anadara kagoshimensis AB854404 Japan
Anadara kagoshimensis AB854402 Japan
Anadara kagoshimensis OL629074
Anadara kagoshimensis OL629075
Anadara kagoshimensis OL629073
Anadara kagoshimensis PQ799215 South Korea
Anadara kagoshimensis PQ799216 South Korea
Anadara kagoshimensis KF417439
Anadara kagoshimensis KP253072
Anadara kagoshimensis AY040553
Anadara kagoshimensis KM267563 Romania Black Sea
Anadara kagoshimensis KM267562 Romania Black Sea
Anadara kagoshimensis KF417435
Anadara kagoshimensis KF417436
Anadara kagoshimensis KP253069
Anadara kagoshimensis MF426975 Italy
Anadara kagoshimensis MF426976 Italy
Anadara kagoshimensis KF417440
Anadara kagoshimensis MF426979 Italy
Anadara kagoshimensis MF426977 Italy
Anadara kagoshimensis MF426978 Italy
Anadara kagoshimensis MF426980 Italy
Anadara kagoshimensis KF417438
Anadara kagoshimensis MF426981 Italy
Anadara kagoshimensis MF426982 Italy
Anadara kagoshimensis MF426983 Italy
Anadara kagoshimensis KF417437
Anadara kagoshimensis MF426984 Italy
Anadara kagoshimensis PQ799182 China
Anadara kagoshimensis KJ490941 Korea
Anadara kagoshimensis KJ490940 Russia Black Sea
Anadara kagoshimensis PQ799183 China
Anadara kagoshimensis KP253073
Anadara antiquata MZ675504
Anadara kagoshimensis HQ258851 China
Anadara kagoshimensis KU341876
Anadara kagoshimensis KU341875
Anadara kagoshimensis KU341874
Anadara kagoshimensis KT266828 Romania Black Sea
Anadara kagoshimensis AB854388 Japan
Anadara kagoshimensis AB854405 Japan
Anadara kagoshimensis KP253066
Anadara kagoshimensis KP253068
Anadara kagoshimensis KP253071
Anadara kagoshimensis KU341877
Anadara kagoshimensis KU341878
Anadara kagoshimensis KU341879
Anadara kagoshimensis KU341880

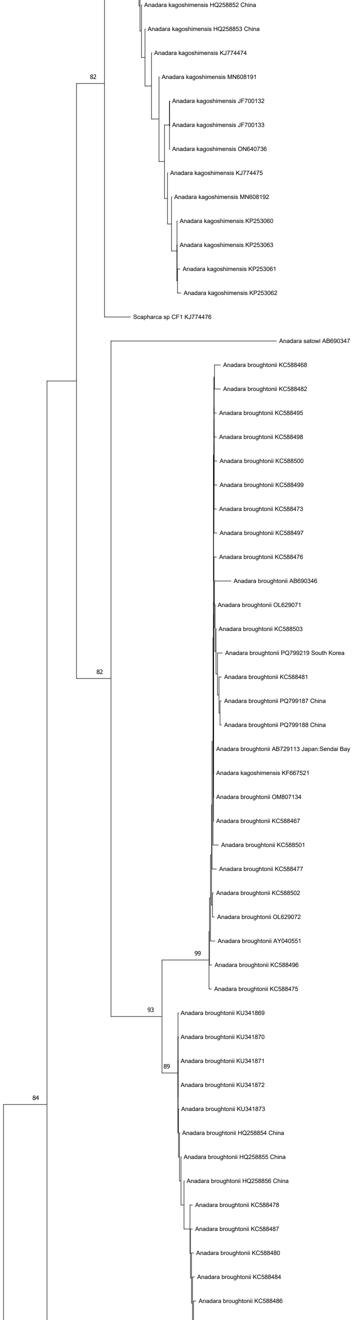
Anadara kagoshimensis HQ258852 China
Anadara kagoshimensis HQ258853 China
Anadara kagoshimensis KJ774474
82
Anadara kagoshimensis MN608191
Anadara kagoshimensis JF700132
Anadara kagoshimensis JF700133
Anadara kagoshimensis ON640736
Anadara kagoshimensis KJ774475
Anadara kagoshimensis MN608192
Anadara kagoshimensis KP253060
Anadara kagoshimensis KP253063
Anadara kagoshimensis KP253061
Anadara kagoshimensis KP253062
Scapharca sp CF1 KJ774476
Anadara satowi AB690347
Anadara broughtonii KC588468
Anadara broughtonii KC588482
Anadara broughtonii KC588495
Anadara broughtonii KC588498
Anadara broughtonii KC588500
Anadara broughtonii KC588499
Anadara broughtonii KC588473
Anadara broughtonii KC588497
Anadara broughtonii KC588476
Anadara broughtonii AB690346
Anadara broughtonii OL629071
Anadara broughtonii KC588503
Anadara broughtonii PQ799219 South Korea
82
Anadara broughtonii KC588481
Anadara broughtonii PQ799187 China
Anadara broughtonii PQ799188 China
Anadara broughtonii AB729113 Japan:Sendai Bay
Anadara kagoshimensis KF667521
Anadara broughtonii OM807134
Anadara broughtonii KC588467
Anadara broughtonii KC588501
Anadara broughtonii KC588477
Anadara broughtonii KC588502
Anadara broughtonii OL629072
Anadara broughtonii AY040551
99
Anadara broughtonii KC588496
Anadara broughtonii KC588475
93
Anadara broughtonii KU341869
Anadara broughtonii KU341870
Anadara broughtonii KU341871
89
Anadara broughtonii KU341872
84
Anadara broughtonii KU341873
Anadara broughtonii HQ258854 China
Anadara broughtonii HQ258855 China
Anadara broughtonii HQ258856 China
Anadara broughtonii KC588478
Anadara broughtonii KC588487
Anadara broughtonii KC588480
Anadara broughtonii KC588484
Anadara broughtonii KC588486

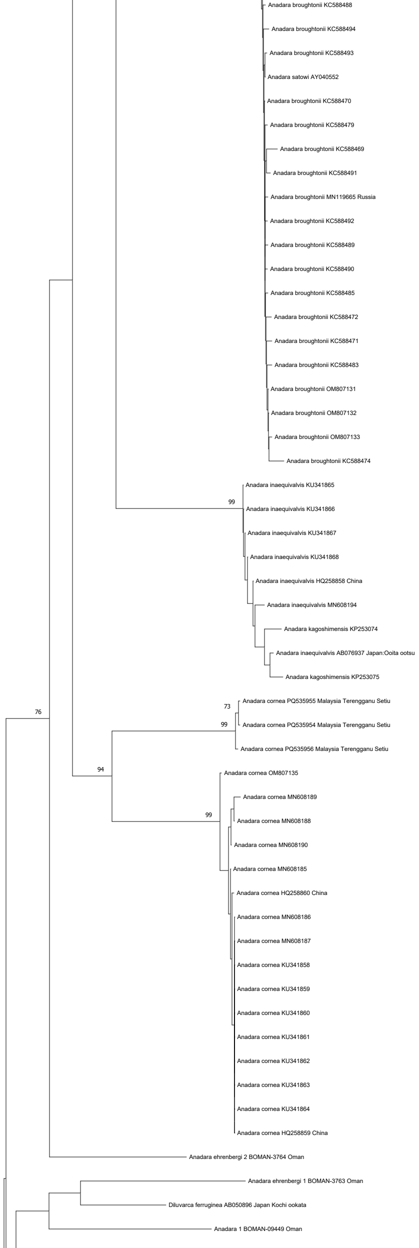

Anadara broughtonii KC588488
Anadara broughtonii KC588494
Anadara broughtonii KC588493
Anadara satowi AY040552
Anadara broughtonii KC588470
Anadara broughtonii KC588479
Anadara broughtonii KC588469
Anadara broughtonii KC588491
Anadara broughtonii MN119665 Russia
Anadara broughtonii KC588492
Anadara broughtonii KC588489
Anadara broughtonii KC588490
Anadara broughtonii KC588485
Anadara broughtonii KC588472
Anadara broughtonii KC588471
Anadara broughtonii KC588483
Anadara broughtonii OM807131
Anadara broughtonii OM807132
Anadara broughtonii OM807133
Anadara broughtonii KC588474
Anadara inaequivalvis KU341865
99
Anadara inaequivalvis KU341866
Anadara inaequivalvis KU341867
Anadara inaequivalvis KU341868
Anadara inaequivalvis HQ258858 China
Anadara inaequivalvis MN608194
Anadara kagoshimensis KP253074
Anadara inaequivalvis AB076937 Japan:Ooita ootsu
Anadara kagoshimensis KP253075
73
Anadara cornea PQ535955 Malaysia Terengganu Setiu
76
99
Anadara cornea PQ535954 Malaysia Terengganu Setiu
Anadara cornea PQ535956 Malaysia Terengganu Setiu
94
Anadara cornea OM807135
Anadara cornea MN608189
99
Anadara cornea MN608188
Anadara cornea MN608190
Anadara cornea MN608185
Anadara cornea HQ258860 China
Anadara cornea MN608186
Anadara cornea MN608187
Anadara cornea KU341858
Anadara cornea KU341859
Anadara cornea KU341860
Anadara cornea KU341861
Anadara cornea KU341862
Anadara cornea KU341863
Anadara cornea KU341864
Anadara cornea HQ258859 China
Anadara ehrenbergi 2 BOMAN-3764 Oman
Anadara ehrenbergi 1 BOMAN-3763 Oman
Diluvarca ferruginea AB050896 Japan Kochi ookata
Anadara 1 BOMAN-09449 Oman

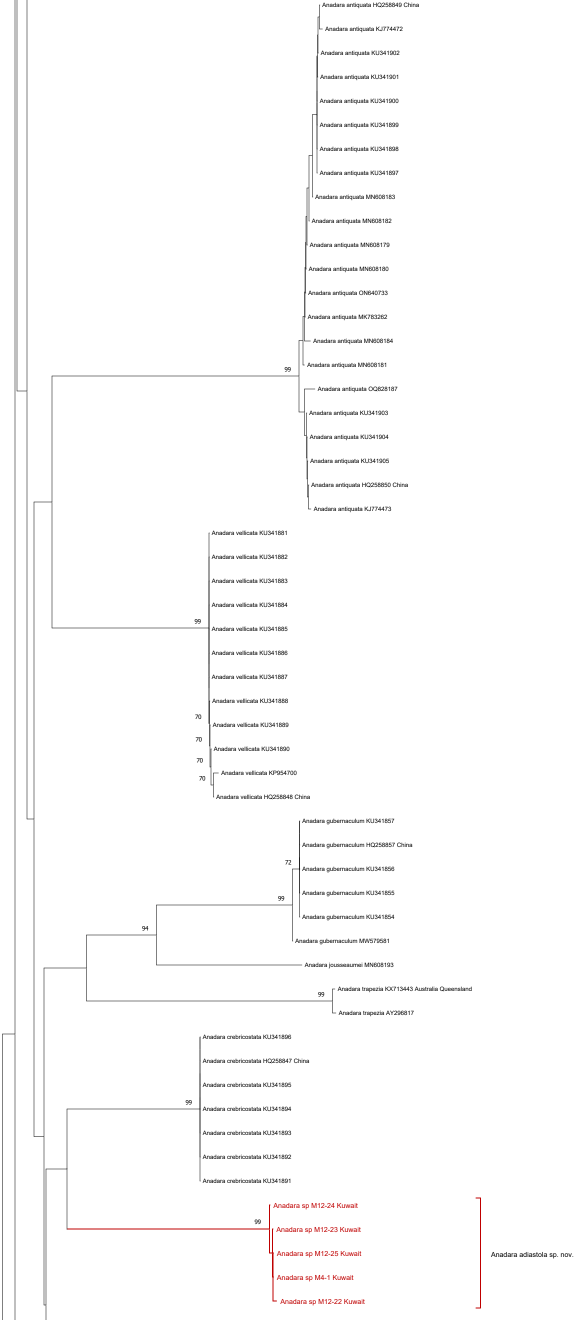
Anadara antiquata HQ258849 China
Anadara antiquata KJ774472
Anadara antiquata KU341902
Anadara antiquata KU341901
Anadara antiquata KU341900
Anadara antiquata KU341899
Anadara antiquata KU341898
Anadara antiquata KU341897
Anadara antiquata MN608183
Anadara antiquata MN608182
Anadara antiquata MN608179
Anadara antiquata MN608180
Anadara antiquata ON640733
Anadara antiquata MK783262
Anadara antiquata MN608184
99
Anadara antiquata MN608181
Anadara antiquata OQ828187
Anadara antiquata KU341903
Anadara antiquata KU341904
Anadara antiquata KU341905
Anadara antiquata HQ258850 China
Anadara antiquata KJ774473
Anadara vellicata KU341881
Anadara vellicata KU341882
Anadara vellicata KU341883
Anadara vellicata KU341884
99
Anadara vellicata KU341885
Anadara vellicata KU341886
Anadara vellicata KU341887
Anadara vellicata KU341888
70
Anadara vellicata KU341889
70
Anadara vellicata KU341890
70
Anadara vellicata KP954700
70
Anadara vellicata HQ258848 China
Anadara gubernaculum KU341857
Anadara gubernaculum HQ258857 China
72
Anadara gubernaculum KU341856
99
Anadara gubernaculum KU341855
94
Anadara gubernaculum KU341854
Anadara gubernaculum MW579581
Anadara jousseaumei MN608193
Anadara trapezia KX713443 Australia Queensland
99
Anadara trapezia AY296817
Anadara crebricostata KU341896
Anadara crebricostata HQ258847 China
Anadara crebricostata KU341895
99
Anadara crebricostata KU341894
Anadara crebricostata KU341893
Anadara crebricostata KU341892
Anadara crebricostata KU341891
Anadara sp M12-24 Kuwait
99
Anadara sp M12-23 Kuwait
Anadara sp M12-25 Kuwait
Anadara adiastola sp. nov.
Anadara sp M4-1 Kuwait
Anadara sp M12-22 Kuwait

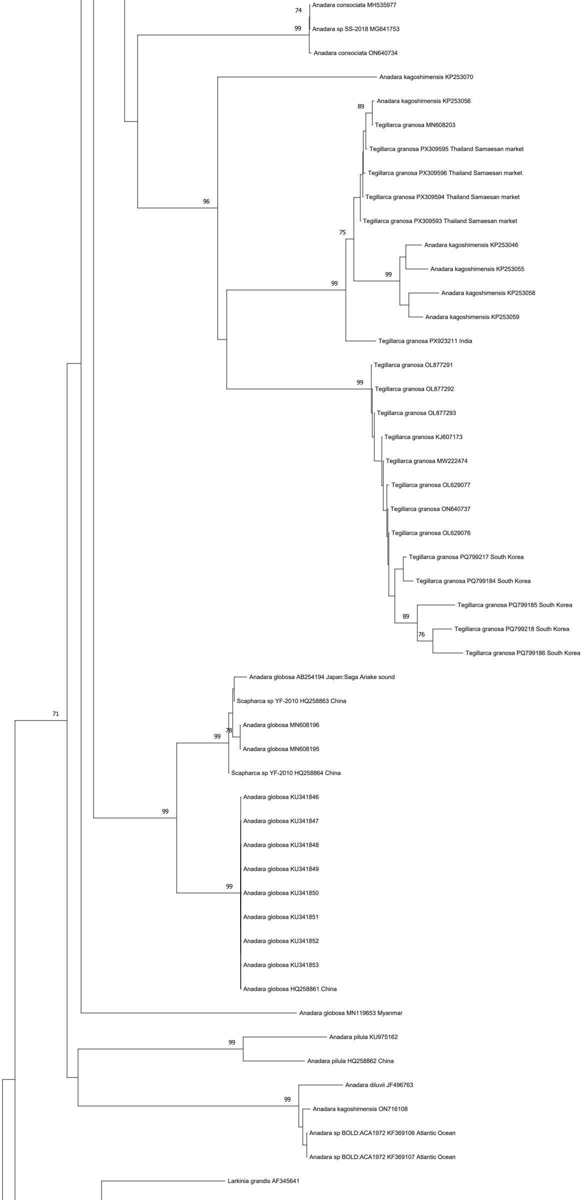

Anadara consociata MH535977
Anadara sp SS-2018 MG641753
Anadara consociata ON640734
Anadara kagoshimensis KP253070
Anadara kagoshimensis KP253056
Tegillarca granosa MN608203
Tegillarca granosa PX309595 Thailand Samaesan market
Tegillarca granosa PX309596 Thailand Samaesan market
Tegillarca granosa PX309594 Thailand Samaesan market
Tegillarca granosa PX309593 Thailand Samaesan market
Anadara kagoshimensis KP253046
Anadara kagoshimensis KP253055
Anadara kagoshimensis KP253058
Anadara kagoshimensis KP253059
Tegillarca granosa PX923211 India
Tegillarca granosa OL877291
Tegillarca granosa OL877292
Tegillarca granosa OL877293
Tegillarca granosa KJ607173
Tegillarca granosa MW222474
Tegillarca granosa OL629077
Tegillarca granosa ON640737
Tegillarca granosa OL629076
Tegillarca granosa PQ799217 South Korea
Tegillarca granosa PQ799184 South Korea
Tegillarca granosa PQ799185 South Korea
Tegillarca granosa PQ799218 South Korea
Tegillarca granosa PQ799186 South Korea
Anadara globosa AB254194 Japan:Saga Ariake sound
Scapharca sp YF-2010 HQ258863 China
Anadara globosa MN608196
Anadara globosa MN608195
Scapharca sp YF-2010 HQ258864 China
Anadara globosa KU341846
Anadara globosa KU341847
Anadara globosa KU341848
Anadara globosa KU341849
Anadara globosa KU341850
Anadara globosa KU341851
Anadara globosa KU341852
Anadara globosa KU341853
Anadara globosa HQ258861 China
Anadara globosa MN119653 Myanmar
Anadara pilula KU975162
Anadara pilula HQ258862 China
Anadara diluvii JF496763
Anadara kagoshimensis ON716108
Anadara sp BOLD:ACA1972 KF369106 Atlantic Ocean
Anadara sp BOLD:ACA1972 KF369107 Atlantic Ocean
Larkinia grandis AF345641

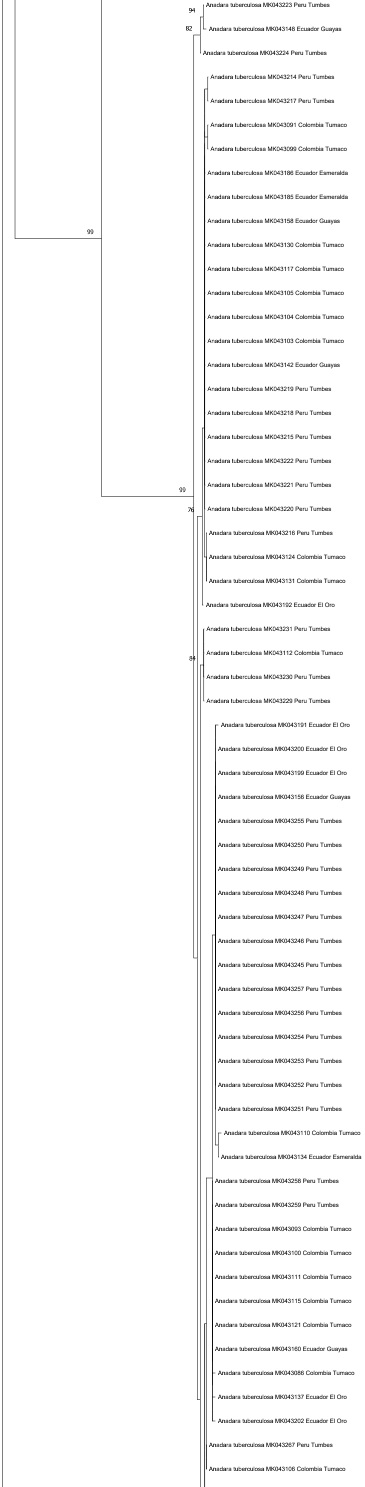
94
Anadara tuberculosa MK043223 Peru Tumbes
82
Anadara tuberculosa MK043148 Ecuador Guayas
Anadara tuberculosa MK043224 Peru Tumbes
Anadara tuberculosa MK043214 Peru Tumbes
Anadara tuberculosa MK043217 Peru Tumbes
Anadara tuberculosa MK043091 Colombia Tumaco
Anadara tuberculosa MK043099 Colombia Tumaco
Anadara tuberculosa MK043186 Ecuador Esmeralda
Anadara tuberculosa MK043185 Ecuador Esmeralda
Anadara tuberculosa MK043158 Ecuador Guayas
99
Anadara tuberculosa MK043130 Colombia Tumaco
Anadara tuberculosa MK043117 Colombia Tumaco
Anadara tuberculosa MK043105 Colombia Tumaco
Anadara tuberculosa MK043104 Colombia Tumaco
Anadara tuberculosa MK043103 Colombia Tumaco
Anadara tuberculosa MK043142 Ecuador Guayas
Anadara tuberculosa MK043219 Peru Tumbes
Anadara tuberculosa MK043218 Peru Tumbes
Anadara tuberculosa MK043215 Peru Tumbes
Anadara tuberculosa MK043222 Peru Tumbes
Anadara tuberculosa MK043221 Peru Tumbes
99
76
Anadara tuberculosa MK043220 Peru Tumbes
Anadara tuberculosa MK043216 Peru Tumbes
Anadara tuberculosa MK043124 Colombia Tumaco
Anadara tuberculosa MK043131 Colombia Tumaco
Anadara tuberculosa MK043192 Ecuador El Oro
Anadara tuberculosa MK043231 Peru Tumbes
84
Anadara tuberculosa MK043112 Colombia Tumaco
Anadara tuberculosa MK043230 Peru Tumbes
Anadara tuberculosa MK043229 Peru Tumbes
Anadara tuberculosa MK043191 Ecuador El Oro
Anadara tuberculosa MK043200 Ecuador El Oro
Anadara tuberculosa MK043199 Ecuador El Oro
Anadara tuberculosa MK043156 Ecuador Guayas
Anadara tuberculosa MK043255 Peru Tumbes
Anadara tuberculosa MK043250 Peru Tumbes
Anadara tuberculosa MK043249 Peru Tumbes
Anadara tuberculosa MK043248 Peru Tumbes
Anadara tuberculosa MK043247 Peru Tumbes
Anadara tuberculosa MK043246 Peru Tumbes
Anadara tuberculosa MK043245 Peru Tumbes
Anadara tuberculosa MK043257 Peru Tumbes
Anadara tuberculosa MK043256 Peru Tumbes
Anadara tuberculosa MK043254 Peru Tumbes
Anadara tuberculosa MK043253 Peru Tumbes
Anadara tuberculosa MK043252 Peru Tumbes
Anadara tuberculosa MK043251 Peru Tumbes
Anadara tuberculosa MK043110 Colombia Tumaco
Anadara tuberculosa MK043134 Ecuador Esmeralda
Anadara tuberculosa MK043258 Peru Tumbes
Anadara tuberculosa MK043259 Peru Tumbes
Anadara tuberculosa MK043093 Colombia Tumaco
Anadara tuberculosa MK043100 Colombia Tumaco
Anadara tuberculosa MK043111 Colombia Tumaco
Anadara tuberculosa MK043115 Colombia Tumaco
Anadara tuberculosa MK043121 Colombia Tumaco
Anadara tuberculosa MK043160 Ecuador Guayas
Anadara tuberculosa MK043086 Colombia Tumaco
Anadara tuberculosa MK043137 Ecuador El Oro
Anadara tuberculosa MK043202 Ecuador El Oro
Anadara tuberculosa MK043267 Peru Tumbes
Anadara tuberculosa MK043106 Colombia Tumaco

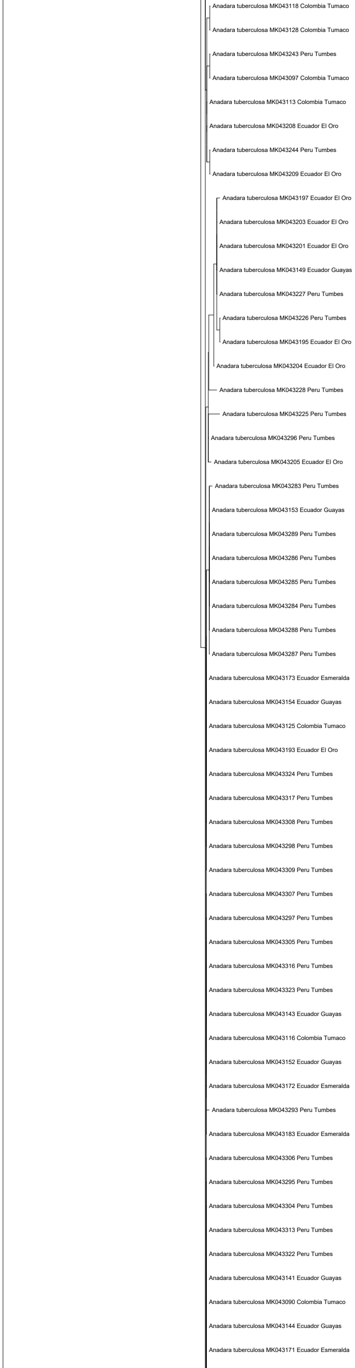
Anadara tuberculosa MK043118 Colombia Tumaco
Anadara tuberculosa MK043128 Colombia Tumaco
Anadara tuberculosa MK043243 Peru Tumbes
Anadara tuberculosa MK043097 Colombia Tumaco
Anadara tuberculosa MK043113 Colombia Tumaco
Anadara tuberculosa MK043208 Ecuador El Oro
Anadara tuberculosa MK043244 Peru Tumbes
Anadara tuberculosa MK043209 Ecuador El Oro
Anadara tuberculosa MK043197 Ecuador El Oro
Anadara tuberculosa MK043203 Ecuador El Oro
Anadara tuberculosa MK043201 Ecuador El Oro
Anadara tuberculosa MK043149 Ecuador Guayas
Anadara tuberculosa MK043227 Peru Tumbes
Anadara tuberculosa MK043226 Peru Tumbes
Anadara tuberculosa MK043195 Ecuador El Oro
Anadara tuberculosa MK043204 Ecuador El Oro
Anadara tuberculosa MK043228 Peru Tumbes
Anadara tuberculosa MK043225 Peru Tumbes
Anadara tuberculosa MK043296 Peru Tumbes
Anadara tuberculosa MK043205 Ecuador El Oro
Anadara tuberculosa MK043283 Peru Tumbes
Anadara tuberculosa MK043153 Ecuador Guayas
Anadara tuberculosa MK043289 Peru Tumbes
Anadara tuberculosa MK043286 Peru Tumbes
Anadara tuberculosa MK043285 Peru Tumbes
Anadara tuberculosa MK043284 Peru Tumbes
Anadara tuberculosa MK043288 Peru Tumbes
Anadara tuberculosa MK043287 Peru Tumbes
Anadara tuberculosa MK043173 Ecuador Esmeralda
Anadara tuberculosa MK043154 Ecuador Guayas
Anadara tuberculosa MK043125 Colombia Tumaco
Anadara tuberculosa MK043193 Ecuador El Oro
Anadara tuberculosa MK043324 Peru Tumbes
Anadara tuberculosa MK043317 Peru Tumbes
Anadara tuberculosa MK043308 Peru Tumbes
Anadara tuberculosa MK043298 Peru Tumbes
Anadara tuberculosa MK043309 Peru Tumbes
Anadara tuberculosa MK043307 Peru Tumbes
Anadara tuberculosa MK043297 Peru Tumbes
Anadara tuberculosa MK043305 Peru Tumbes
Anadara tuberculosa MK043316 Peru Tumbes
Anadara tuberculosa MK043323 Peru Tumbes
Anadara tuberculosa MK043143 Ecuador Guayas
Anadara tuberculosa MK043116 Colombia Tumaco
Anadara tuberculosa MK043152 Ecuador Guayas
Anadara tuberculosa MK043172 Ecuador Esmeralda
Anadara tuberculosa MK043293 Peru Tumbes
Anadara tuberculosa MK043183 Ecuador Esmeralda
Anadara tuberculosa MK043306 Peru Tumbes
Anadara tuberculosa MK043295 Peru Tumbes
Anadara tuberculosa MK043304 Peru Tumbes
Anadara tuberculosa MK043313 Peru Tumbes
Anadara tuberculosa MK043322 Peru Tumbes
Anadara tuberculosa MK043141 Ecuador Guayas
Anadara tuberculosa MK043090 Colombia Tumaco
Anadara tuberculosa MK043144 Ecuador Guayas
Anadara tuberculosa MK043171 Ecuador Esmeralda

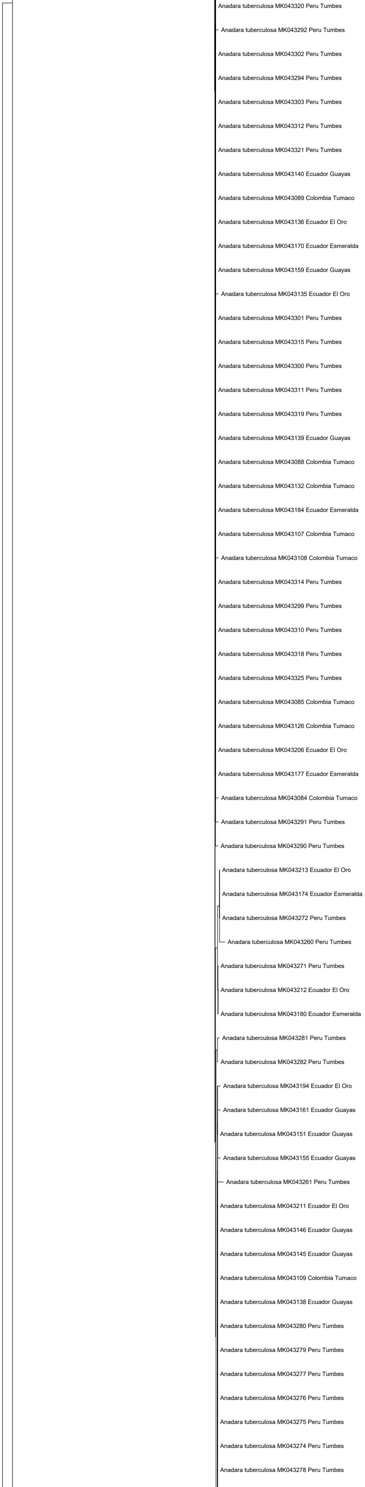
Anadara tuberculosa MK043320 Peru Tumbes
Anadara tuberculosa MK043292 Peru Tumbes
Anadara tuberculosa MK043302 Peru Tumbes
Anadara tuberculosa MK043294 Peru Tumbes
Anadara tuberculosa MK043303 Peru Tumbes
Anadara tuberculosa MK043312 Peru Tumbes
Anadara tuberculosa MK043321 Peru Tumbes
Anadara tuberculosa MK043140 Ecuador Guayas
Anadara tuberculosa MK043089 Colombia Tumaco
Anadara tuberculosa MK043136 Ecuador El Oro
Anadara tuberculosa MK043170 Ecuador Esmeralda
Anadara tuberculosa MK043159 Ecuador Guayas
Anadara tuberculosa MK043135 Ecuador El Oro
Anadara tuberculosa MK043301 Peru Tumbes
Anadara tuberculosa MK043315 Peru Tumbes
Anadara tuberculosa MK043300 Peru Tumbes
Anadara tuberculosa MK043311 Peru Tumbes
Anadara tuberculosa MK043319 Peru Tumbes
Anadara tuberculosa MK043139 Ecuador Guayas
Anadara tuberculosa MK043088 Colombia Tumaco
Anadara tuberculosa MK043132 Colombia Tumaco
Anadara tuberculosa MK043184 Ecuador Esmeralda
Anadara tuberculosa MK043107 Colombia Tumaco
Anadara tuberculosa MK043108 Colombia Tumaco
Anadara tuberculosa MK043314 Peru Tumbes
Anadara tuberculosa MK043299 Peru Tumbes
Anadara tuberculosa MK043310 Peru Tumbes
Anadara tuberculosa MK043318 Peru Tumbes
Anadara tuberculosa MK043325 Peru Tumbes
Anadara tuberculosa MK043085 Colombia Tumaco
Anadara tuberculosa MK043126 Colombia Tumaco
Anadara tuberculosa MK043206 Ecuador El Oro
Anadara tuberculosa MK043177 Ecuador Esmeralda
Anadara tuberculosa MK043084 Colombia Tumaco
Anadara tuberculosa MK043291 Peru Tumbes
Anadara tuberculosa MK043290 Peru Tumbes
Anadara tuberculosa MK043213 Ecuador El Oro
Anadara tuberculosa MK043174 Ecuador Esmeralda
Anadara tuberculosa MK043272 Peru Tumbes
Anadara tuberculosa MK043260 Peru Tumbes
Anadara tuberculosa MK043271 Peru Tumbes
Anadara tuberculosa MK043212 Ecuador El Oro
Anadara tuberculosa MK043180 Ecuador Esmeralda
Anadara tuberculosa MK043281 Peru Tumbes
Anadara tuberculosa MK043282 Peru Tumbes
Anadara tuberculosa MK043194 Ecuador El Oro
Anadara tuberculosa MK043161 Ecuador Guayas
Anadara tuberculosa MK043151 Ecuador Guayas
Anadara tuberculosa MK043155 Ecuador Guayas
Anadara tuberculosa MK043261 Peru Tumbes
Anadara tuberculosa MK043211 Ecuador El Oro
Anadara tuberculosa MK043146 Ecuador Guayas
Anadara tuberculosa MK043145 Ecuador Guayas
Anadara tuberculosa MK043109 Colombia Tumaco
Anadara tuberculosa MK043138 Ecuador Guayas
Anadara tuberculosa MK043280 Peru Tumbes
Anadara tuberculosa MK043279 Peru Tumbes
Anadara tuberculosa MK043277 Peru Tumbes
Anadara tuberculosa MK043276 Peru Tumbes
Anadara tuberculosa MK043275 Peru Tumbes
Anadara tuberculosa MK043274 Peru Tumbes
Anadara tuberculosa MK043278 Peru Tumbes

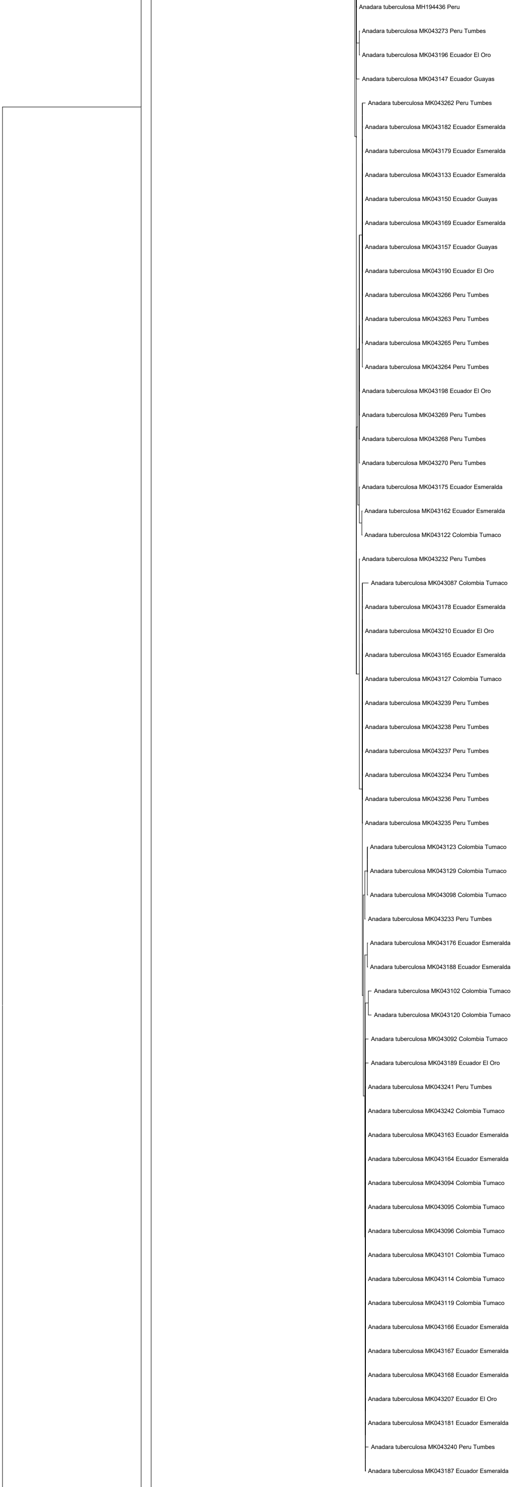

Anadara tuberculosa MH194436 Peru
Anadara tuberculosa MK043273 Peru Tumbes
Anadara tuberculosa MK043196 Ecuador El Oro
Anadara tuberculosa MK043147 Ecuador Guayas
Anadara tuberculosa MK043262 Peru Tumbes
Anadara tuberculosa MK043182 Ecuador Esmeralda
Anadara tuberculosa MK043179 Ecuador Esmeralda
Anadara tuberculosa MK043133 Ecuador Esmeralda
Anadara tuberculosa MK043150 Ecuador Guayas
Anadara tuberculosa MK043169 Ecuador Esmeralda
Anadara tuberculosa MK043157 Ecuador Guayas
Anadara tuberculosa MK043190 Ecuador El Oro
Anadara tuberculosa MK043266 Peru Tumbes
Anadara tuberculosa MK043263 Peru Tumbes
Anadara tuberculosa MK043265 Peru Tumbes
Anadara tuberculosa MK043264 Peru Tumbes
Anadara tuberculosa MK043198 Ecuador El Oro
Anadara tuberculosa MK043269 Peru Tumbes
Anadara tuberculosa MK043268 Peru Tumbes
Anadara tuberculosa MK043270 Peru Tumbes
Anadara tuberculosa MK043175 Ecuador Esmeralda
Anadara tuberculosa MK043162 Ecuador Esmeralda
Anadara tuberculosa MK043122 Colombia Tumaco
Anadara tuberculosa MK043232 Peru Tumbes
Anadara tuberculosa MK043087 Colombia Tumaco
Anadara tuberculosa MK043178 Ecuador Esmeralda
Anadara tuberculosa MK043210 Ecuador El Oro
Anadara tuberculosa MK043165 Ecuador Esmeralda
Anadara tuberculosa MK043127 Colombia Tumaco
Anadara tuberculosa MK043239 Peru Tumbes
Anadara tuberculosa MK043238 Peru Tumbes
Anadara tuberculosa MK043237 Peru Tumbes
Anadara tuberculosa MK043234 Peru Tumbes
Anadara tuberculosa MK043236 Peru Tumbes
Anadara tuberculosa MK043235 Peru Tumbes
Anadara tuberculosa MK043123 Colombia Tumaco
Anadara tuberculosa MK043129 Colombia Tumaco
Anadara tuberculosa MK043098 Colombia Tumaco
Anadara tuberculosa MK043233 Peru Tumbes
Anadara tuberculosa MK043176 Ecuador Esmeralda
Anadara tuberculosa MK043188 Ecuador Esmeralda
Anadara tuberculosa MK043102 Colombia Tumaco
Anadara tuberculosa MK043120 Colombia Tumaco
Anadara tuberculosa MK043092 Colombia Tumaco
Anadara tuberculosa MK043189 Ecuador El Oro
Anadara tuberculosa MK043241 Peru Tumbes
Anadara tuberculosa MK043242 Colombia Tumaco
Anadara tuberculosa MK043163 Ecuador Esmeralda
Anadara tuberculosa MK043164 Ecuador Esmeralda
Anadara tuberculosa MK043094 Colombia Tumaco
Anadara tuberculosa MK043095 Colombia Tumaco
Anadara tuberculosa MK043096 Colombia Tumaco
Anadara tuberculosa MK043101 Colombia Tumaco
Anadara tuberculosa MK043114 Colombia Tumaco
Anadara tuberculosa MK043119 Colombia Tumaco
Anadara tuberculosa MK043166 Ecuador Esmeralda
Anadara tuberculosa MK043167 Ecuador Esmeralda
Anadara tuberculosa MK043168 Ecuador Esmeralda
Anadara tuberculosa MK043207 Ecuador El Oro
Anadara tuberculosa MK043181 Ecuador Esmeralda
Anadara tuberculosa MK043240 Peru Tumbes
Anadara tuberculosa MK043187 Ecuador Esmeralda

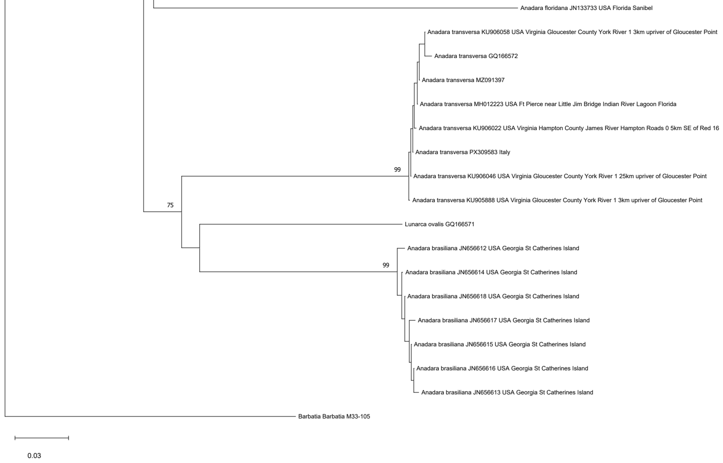
Anadara floridana JN133733 USA Florida Sanibel
Anadara transversa KU906058 USA Virginia Gloucester County York River 1 3km upriver of Gloucester Point
Anadara transversa GQ166572
Anadara transversa MZ091397
Anadara transversa MH012223 USA Ft Pierce near Little Jim Bridge Indian River Lagoon Florida
Anadara transversa KU906022 USA Virginia Hampton County James River Hampton Roads 0 5km SE of Red 16
Anadara transversa PX309583 Italy
99
Anadara transversa KU906046 USA Virginia Gloucester County York River 1 25km upriver of Gloucester Point
75
Anadara transversa KU905888 USA Virginia Gloucester County York River 1 3km upriver of Gloucester Point
Lunarca ovalis GQ166571
Anadara brasiliana JN656612 USA Georgia St Catherines Island
99
Anadara brasiliana JN656614 USA Georgia St Catherines Island
Anadara brasiliana JN656618 USA Georgia St Catherines Island
Anadara brasiliana JN656617 USA Georgia St Catherines Island
Anadara brasiliana JN656615 USA Georgia St Catherines Island
Anadara brasiliana JN656616 USA Georgia St Catherines Island
Anadara brasiliana JN656613 USA Georgia St Catherines Island
Barbatia Barbatia M33-105
0.03
